# Direct observation of degassing during decompression of basaltic magma

**DOI:** 10.1126/sciadv.ado2585

**Published:** 2024-08-16

**Authors:** Barbara Bonechi, Margherita Polacci, Fabio Arzilli, Giuseppe La Spina, Jean-Louis Hazemann, Richard A. Brooker, Robert Atwood, Sebastian Marussi, Peter D. Lee, Roy A. Wogelius, Jonathan Fellowes, Mike R. Burton

**Affiliations:** ^1^ Department of Earth and Environmental Sciences, University of Manchester, Manchester, UK.; ^2^ School of Science and Technology, Geology Division, University of Camerino, Camerino, Italy.; ^3^ Istituto Nazionale di Geofisica e Vulcanologia, Osservatorio Etneo, Catania, Italy.; ^4^ Université Grenoble Alpes, CNRS, Grenoble INP, Institut Néel, Grenoble, France.; ^5^ School of Earth Sciences, University of Bristol, Bristol, UK.; ^6^ Diamond Light Source, Harwell Science and Innovation Campus, Harwell, Oxfordshire, UK.; ^7^ Department of Mechanical Engineering, University College London, London, UK.; ^8^ Research Complex at Harwell, Rutherford Appleton Laboratory, Harwell, Oxfordshire, UK.

## Abstract

Transitions in eruptive style during volcanic eruptions strongly depend on how easily gas and magma decouple during ascent. Stronger gas-melt coupling favors highly explosive eruptions, whereas weaker coupling promotes lava fountaining and lava flows. The mechanisms producing these transitions are still poorly understood because of a lack of direct observations of bubble dynamics under natural magmatic conditions. Here, we combine x-ray radiography with a novel high-pressure/high-temperature apparatus to observe and quantify in real-time bubble growth and coalescence in basaltic magmas from 100 megapascals to surface. For low-viscosity magmas, bubbles coalesce and recover a spherical shape within 3 seconds, implying that, for lava fountaining activity, gas and melt remain coupled during the ascent up to the last hundred meters of the conduit. For higher-viscosity magmas, recovery times become longer, promoting connected bubble pathways. This apparatus opens frontiers in unraveling magmatic/volcanic processes, leading to improved hazard assessment and risk mitigation.

## Teaser

Direct observation of degassing and bubble coalescence in magmas reveals processes that control the style of volcanic eruptions.

## INTRODUCTION

Volatile exsolution, expansion, and outgassing during magma ascent play a key control on the intensity and style of eruptions (*1*). The vesiculation process is a consequence of the supersaturation of dissolved volatiles within magma (caused, for example, by decreasing pressure during magma ascent or by magma crystallization), producing volatile exsolution (*2*–*4*). Bubbles of initially supercritical fluid, mostly containing water and lower amounts of carbon dioxide, halogens, sulfur, and other volatiles, nucleate and grow during magma ascent (*5*). During ascent, multiple processes occur: decompression-induced nucleation and growth of bubbles, coalescence of these bubbles with one another, and potentially outgassing, through channels formed by a network of coalescing bubbles (*6*–*8*). Ultimately, all of these processes play major roles in determining the eruptive style by influencing whether magma explosively fragments, produces lava fountains, or erupts effusively in lava flows (*7*, *9*, *10*). Understanding bubble coalescence is therefore crucial, given its role in controlling permeability (*11*, *12*), the amount and rate of outgassing (*13*), and hence eruption dynamics (*7*).

As bubbles nucleate and grow, whether during eruptive magma ascent or experiments in the laboratory, interbubble melt films are thinned to the point of rupture, and bubble coalescence starts to occur (*7*, *14*–*20*). Bubble coalescence in volcanology has been investigated by several studies, mainly focusing on the mechanisms that lead toward coalescence (*7*, *15*, *21*–*28*). In general, coalescence is a function of the viscosity of the surrounding melt, and it is an important process affecting bubble dynamics and, hence, magma ascent and transport to the surface (*13*). Bubble coalescence rates (i.e., how many times coalescence events occur per unit time) depend on the timescale of approach between two bubbles (*29*), thinning of the melt film separating two bubbles to a critical value, film rupture, and relaxation (*21*, *22*, *27*, *28*, *30*). Several studies focused on estimating the value of the critical film thickness in silicate melts (*21*, *31*), as well as the timescales of film rupture once the critical thickness is reached (*21*, *22*, *25*–*28*, *30*–*32*). The estimated rupture timescales range from 1 to 10^4^ s, as a function of melt viscosity, bubble size, and the critical film thickness (*21*, *30*). Bubble deformation plays also a key role on coalescence rate and on bubble population (*23*, *24*, *33*), and as a result, they can affect magma ascent dynamics. Other mathematical and numerical studies investigated more closely the dynamics of two merging bubbles in a viscous fluid, showing that the Ohnesorge number (a dimensionless number that relates the viscous forces to inertial and surface tension forces) plays a critical role on how quickly two merged bubbles recover the spherical shape (*34*–*37*). These studies, however, are not calibrated for magmatic fluids; thus, more studies are necessary to constrain these dynamics for volcanic systems.

Following the pioneering study of Sparks (*1*), degassing and bubble dynamics in silicic melts have been widely investigated by means of experiments (*38*–*42*) and numerical models (*20*, *43*–*47*). Many studies have focused on the effect of decompression rate on bubble nucleation in silicic melts (*48*–*54*), while others on the parameters controlling bubble growth (*1*, *16*, *38*, *43*, *45*, *55*, *56*). Accurate insights into the exsolution process were obtained with experiments performed to study vesiculation in silicic melts at both 1 atm (*42*, *55*, *57*, *58*) and high pressure (*39*, *41*, *59*). Recently, further developments concerned the use of in situ four-dimensional (4D) x-ray tomographic microscopy (where sequences of 3D tomographic scans are collected rapidly and continuously, creating a time series of 3D scans) to study vesiculation of silicate melts at high temperature, but at atmospheric pressure (*60*, *61*). However, none of these studies were able to investigate the vesiculation process in basaltic melts in real time at pressures and temperatures comparable to those of an ascending basaltic magma from depth. Here, we combined x-ray synchrotron radiography with a novel x-ray transparent Internally Heated Pressure Vessel (IHPV) apparatus to simulate magma storage and ascent within the crust at pressures ≤100 MPa and temperatures ≤1180°C. With this apparatus, we performed in situ vesiculation experiments to study bubble growth and coalescence in a basaltic magma in real time at magmatic pressures and temperatures under water-saturated conditions.

Our experiments provide visualization and quantification of timescales of bubble formation (i.e., bubble growth, expansion, and coalescence) in real time, confirming and empirically validating theoretical and modeling results for bubble growth and expansion. The experimental results offer an improved understanding of coupling and decoupling between magma and volatiles during ascent in the conduit, providing insights into processes leading to eruptive style transitions and, ultimately, having fundamental implications for hazard assessment and risk mitigation in areas of active basaltic volcanism. Although basaltic volcanoes are usually characterized by effusive and mildly explosive Strombolian and lava fountaining activity (*8*), some basaltic volcanoes also produce highly explosive Plinian eruptions (*62*–*68*), with a much higher risk for population safety and critical infrastructure, as well as larger environmental impacts.

## RESULTS AND DISCUSSION

### High-pressure, high-temperature x-ray radiography experiments

Experiments were performed in situ at beamline I12-JEEP, Diamond Light Source, Harwell, UK, combining a novel x-ray transparent IHPV apparatus (fig. S1) with fast synchrotron x-ray radiography. We used a hydrous basaltic glass (table S1) from the 2001 Mt. Etna eruption as the starting material (see Materials and Methods).

We performed decompression experiments at superliquidus and subliquidus conditions to study the vesiculation process at different viscosities and crystallinities. To investigate vesiculation kinetics in pure basaltic melts with 1 wt % of H_2_O dissolved, we performed three decompression experiments at superliquidus (Superliq_Dec) conditions (i.e., no crystals) with decompression rates of 0.05 and 0.08 MPa s^−1^ representative of basaltic magma ascent rates during fountaining activity (*69*). The temperature was kept constant at 1180°C during decompression, above the bulk liquidus that is represented by clinopyroxene (table S2). To investigate vesiculation kinetics in crystal-bearing basaltic melts with 0.5, 1, and 2 wt % dissolved H_2_O, we performed five decompression experiments at subliquidus conditions (Subliq_Dec). This set of experiments is characterized by an initial cooling, with a cooling rate of 0.75°C s^−1^, at isobaric conditions (50 and 75 MPa) from 1180°C to different target temperatures (1050° to 1080°C) to promote crystallization before decompression. After subliquidus conditions were reached, the system was isothermally decompressed to 0.1 MPa with a decompression rate of 0.08 MPa s^−1^ to simulate different magma ascent rates during basaltic explosive or fountaining activities (*69*).

An additional decompression experiment at superliquidus condition was performed to investigate vesiculation kinetics in a hydrous rhyolitic melt (with 0.2 wt % of H_2_O); this experiment gives us the opportunity to compare vesiculation kinetics of basaltic and rhyolitic melts and to extrapolate the role of viscosity and crystals on bubble kinetics and dynamics. In particular, this allows us to compare experiments with a high-viscosity crystal-free magma (rhyolite at superliquidus temperature) with those with a less viscous melt but with similar bulk viscosity, due to the presence of crystals (Subliq_Dec experiments). Once all the experiments performed reached 0.1 MPa, the temperature was dropped to the atmospheric one with a continuous cooling of 0.75°C s^−1^ (fig. S2).

Radiographic images show that the vesiculation process of hydrous basaltic melts is quite different in the Superliq_Dec and the Subliq_Dec experiments (table S2). In the first group, we observe the growth of single bubbles that during the decompression paths tend to coalesce forming an individual bubble moving upwards to the top of the system setup (movies S1A and S2). A different behavior is observed in the rhyolite sample (Rhyo) in which bubbles remain confined in the melt, reflecting the higher viscosity compared with basalt. In this case, bubbles expand but do not separate from the melt as individual bubbles because of the higher viscosity (movie S1B). The Subliq_Dec experiments behave like the Rhyo as a consequence of the initial cooling (0.75°C s^−1^) that favors crystallization of microlites and a consequent increase in viscosity. In these runs, bubble expansion remains confined in the melt (movies S1C and S3), and outgassing is clearly observed through channels of interconnected bubbles, with a “breathing” pattern where groups of bubbles expand and then release gas through a pathway. While in the Superliq_Dec experiments it is possible to observe the nucleation of spherical bubbles that can easily grow and coalesce, in the Rhyo and Subliq_Dec samples, instead, bubbles are always deformed (e.g., bubbles repulsing each other to accommodate their volume increase), and coalescence occurs but to a lesser extent. In the Subliq_Dec samples, the presence of microlites seems to have a relevant impact on bubble behavior, because bubble expansion occurs following a less regular pattern than that observed in the Rhyo sample (movie S1), in which crystals are absent (fig. S6) because of its superliquidus conditions. The irregular bubble expansion seems dictated by the presence of microlites that physically impede bubble growth confining expansion in the less crystalline portion of the melt. The differences observed in the radiographic images are also visible in the textures of the recovered samples (see Supplementary Text and table S3).

### High–temporal and high–spatial resolution bubble kinetics in basaltic magma

**Fig. 1.**
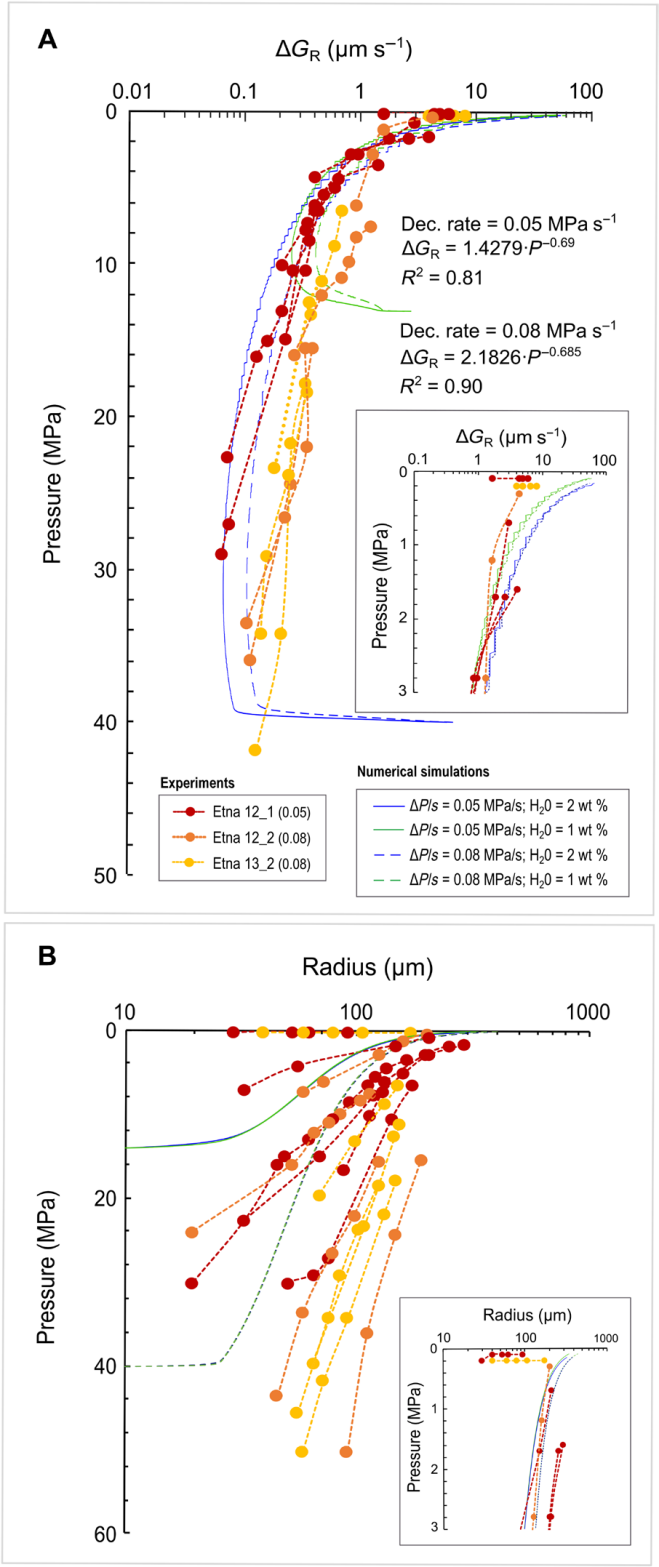
Variation of incremental bubble growth rate and bubble radius with pressure. Plots showing (**A**) incremental bubble growth rate (Δ*G*_R_) and (**B**) bubble radius versus pressure for the Superliq_Dec experiments. In (A), the decompression rate (megapascal per second) for each run is reported in parentheses. Insets show magnification of Δ*G*_R_ (A) and bubble radius (B) at low pressure (*P* < 3 MPa). Both (A) and (B) show the comparison between the observed bubble growth rates and radius measured from the decompression experiments with those calculated (table S5) using the numerical bubble growth model of Coumans *et al.* (*44*). Numerical results are obtained assuming different decompression rates (0.05 and 0.08 MPa s^−1^) and different volatile contents (1 and 2 wt % of H_2_O). Plotted numerical simulations have been computed using the Etna composition (table S1), a magma density of 2700 kg m^−3^, and assuming *N*_b_ = 10^12^ m^−3^.

Our x-ray radiography experiments provided high–temporal and high–spatial resolution bubble kinetics (growth, expansion, and coalescence) in basaltic magma during decompression, simulating magma ascent in volcanic conduits. Once nucleated, bubbles grow by the combined effect of H_2_O diffusion from melt to bubble and decompression-induced gas expansion (*16*). Incremental bubble growth rate (Δ*G*_R_ = Δ*r*/Δ*t*, μm s^−1^) was calculated in the basaltic runs of the Superliq_Dec group as the incremental increase in bubble radius (Δ*r*) over time (Δ*t*) (table S4). We measured the growth rate of bubbles that do not coalesce. In Fig. 1A, we plotted the evolution of Δ*G*_R_, as function of pressure for each of the Superliq_Dec experiments. Each dot represents Δ*G*_R_ calculated for a given bubble, and the dashed lines indicate how Δ*G*_R_ evolves as function of pressure for that specific bubble. Different dashed lines indicate the evolution of Δ*G*_R_ for different bubbles. We observed that, at a given decompression rate, bubble growth rate increases with decreasing pressure (megapascals) following a power-law relation

$$∆G_{R}=1.428\cdot P^{\left(-0.69\right)}\;\text{for a decompression rate of 0.08 MPa}\;s^{-1}$$

$$∆G_{R}=2.183\cdot P^{\left(-0.685\right)}\;\text{for a decompression rate of 0.05 MPa}\;s^{-1}$$

As shown in Fig. 1A, it is possible to see that the Δ*G*_R_ vary from ~10^−1^ to ~10 μm s^−1^ passing from ~40 to 0.1 MPa, with a major rapid increase starting from ~10 MPa. Similarly to Fig. 1A, in Fig. 1B, we plotted the evolution of bubble radius as function of pressure for each of the Superliq_Dec experiments. Also in this case, each dot represents a bubble radius calculated for a given bubble, and the dashed lines indicate how the radius of a specific bubble evolves as function of pressure. Different dashed lines indicate the evolution of radii for different bubbles. Looking at the results, the bubble radius displays a similar trend as that observed for the bubble growth rate (Fig. 1B). In particular, for bubbles grown at different times during the decompression path, we noted that both bubble radius and growth rate increase more gently for bubbles nucleated at the beginning of the decompression (*P* = 30 to 50 MPa; Δ*G*_R_ from ~0.1 to ~0.2 μm s^−1^ in 10 MPa), while they increase faster with a high slope for those nucleated at the end of the decompression path (*P* = 0.1 MPa; Δ*G*_R_ from ~2 to ~8 μm s^−1^ in 0.1 MPa). This can be related to the effect of both the overpressure of bubbles compared to the pressure of the melt and the oversaturation of volatiles. The effect of both bubble overpressure and volatile oversaturation on bubble growth can be noted also comparing our data with those obtained by Masotta *et al.* (*58*) for bubbles grown in a basaltic melt at constant ambient pressure. We noticed that the values they obtained (*G*_R_ = ~10^−1^ to 10^−3^ μm s^−1^) are approximately one to three orders of magnitude lower than those calculated in our experiments once bubbles reached ambient pressure (Δ*G*_R_ ~ 10 μm s^−1^). Masotta *et al.* (*58*) observed fast bubble growth (*G*_R_ = ~10^−1^ to 10 μm s^−1^), triggered by melt degassing, shortly after nucleation (*t* < 20 s), followed by a nearly linear growth (*G*_R_ = ~10^−3^ to 10^−1^ μm s^−1^) for the rest of the experiment (*t* > 20 s). According to Masotta *et al.* (*58*), the fast initial growth, which they did not see directly during their in situ experiments because of the opacity of the sample, is consistent with the classical formulation of bubble growth with the bubble radius proportional to the square root of time (*1*, *45*, *47*) or the logarithmic growth law (*39*, *41*). Thus, the *G*_R_ calculated in this study once bubbles reached ambient pressure (Δ*G*_R_ ~ 10 μm s^−1^), which we measured for time intervals <20 s (table S4), are representative of the *G*_R_ at the very beginning of the exsolution process as reported by Masotta *et al.* (*58*). Another interesting comparison can be done with the bubble growth rates obtained by Bai *et al.* (*70*) for a basaltic melt through 1-atm in situ degassing experiments in which bubble growth is controlled by diffusion of the volatiles from the supersaturated melt to the bubble at constant pressure. The bubble growth rates, which we extrapolated from Bubble Size Distribution data reported in Bai *et al.* (*70*), show values between 10^−2^ and 10^0^ μm s^−1^.

We also compared our experimentally derived growth rates with those calculated using the experimentally validated numerical model of bubble growth by Coumans *et al.* (*44*). This bubble growth model is based on the mathematical formulation of Blower *et al.* (*11*), Proussevitch *et al.* (*31*), and Proussevitch and Sahagian (*20*), and describes bubble expansion in a viscous magma due to decompression and diffusion. This model requires information on the bubble number density (*N*_b_), which is not accessible from our radiography experiments. We performed some numerical simulations using Coumans’ bubble growth model, implementing it with the water diffusion model for basaltic melts (equation 22) of Zhang and Ni (*71*) and assuming common bubble number densities for basaltic magmas (*N*_b_ = 10^10^ to 10^13^ m^−3^) (*54*). We considered two decompression rates (0.05 and 0.08 MPa s^−1^) and two different water content (1 and 2 wt % of H_2_O). The results of these numerical simulations for *N*_b_ = 10^12^ m^−3 ^are plotted in Fig. 1 (blue and green, dashed and solid lines), and they show a good agreement with our observed growth rates except at low pressure (<1 MPa), where modeled growth rates exceed 10 μm s^−1^ (Fig. 1A, fig. S3, and table S5). However, Coumans *et al.* (*44*) reported that the numerical model overestimates bubble growth rates at high gas volume fraction (>0.4), which would explain the discrepancy with the observed values at low pressures. In addition, our growth rates at very low pressures (<1 MPa) might be underestimated because of the effect of the walls of the crucible, which exert a resistance on the melt to deform and flow as bubbles expand, resulting in a reduced expansion of bubbles.

**Fig. 2.**
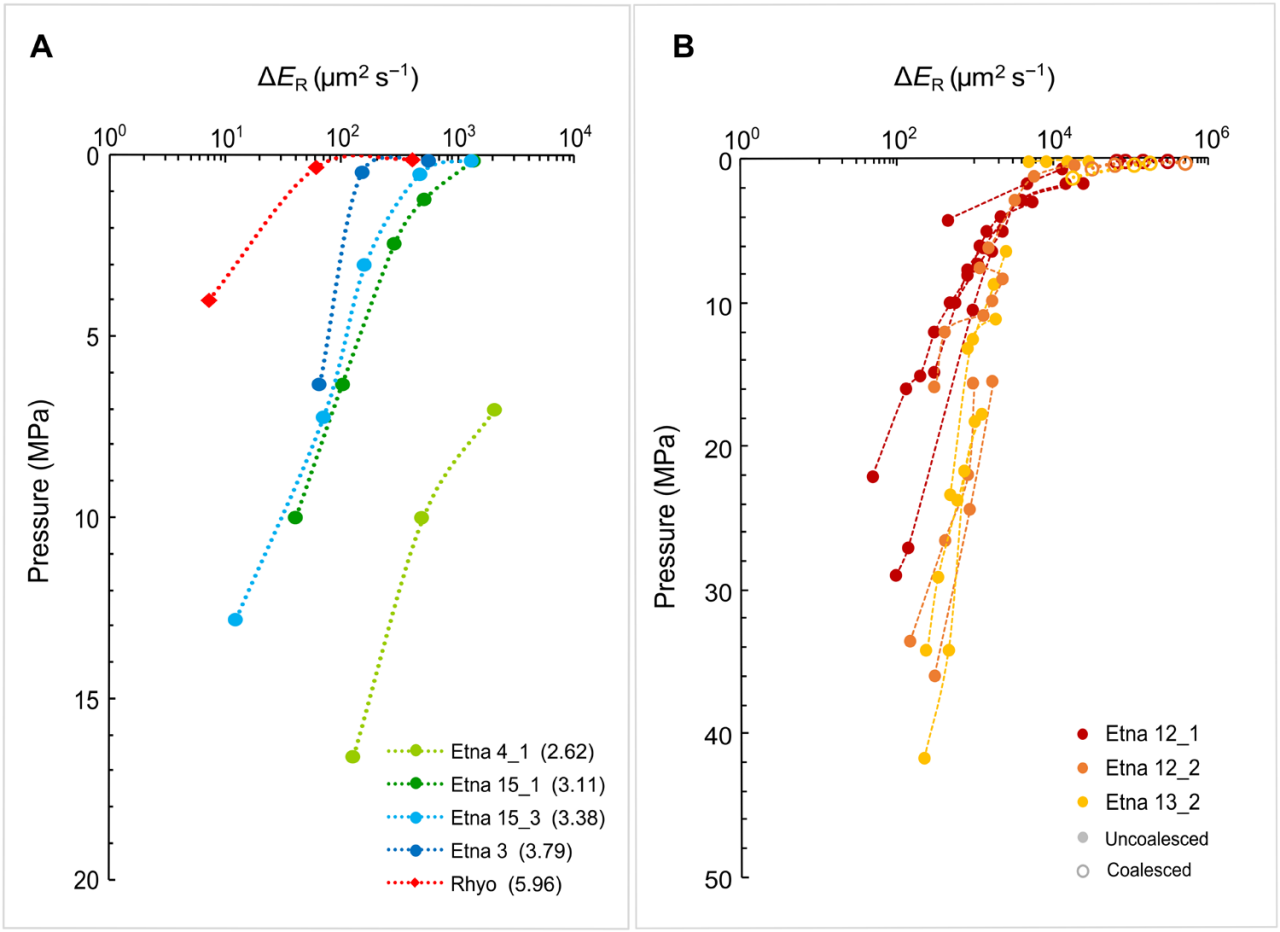
Variation of incremental expansion rate with pressure. Plot showing incremental expansion rate (Δ*E*_R_) versus pressure for (**A**) Subliq_Dec and (**B**) Superliq_Dec experiments. In (A), the liquid viscosity (log Pa·s) for each run is reported in parentheses.

In the Subliq_Dec runs and in the Rhyo sample, bubble growth and coalescence are hampered by the higher viscosity of the melt and not easily resolvable in the radiographic images. Consequently, we could not extrapolate any quantitative data on these processes. However, because the radiographic images show an expansion of the trapped bubbles, it was possible to obtain the incremental bubble expansion rate (Δ*E*_R_ = Δ*A*/Δ*t*; square micrometer per second) as the incremental increase of the bubble area (Δ*A*) with time (Δ*t*). For comparison, we calculated bubble Δ*E*_R_ also in the Superliq_Dec runs that show values approximately one to three orders of magnitude higher than those of the Subliq_Dec ones as a consequence of their different viscosity before the decompression path (Fig. 2 and table S6; see also Supplementary Text for more details).

**Fig. 3.**
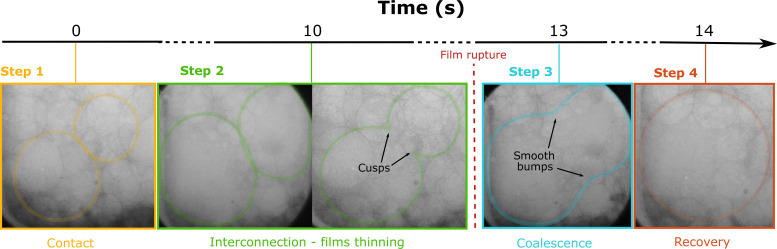
Radiographic images of Superliq_Dec runs showing coalescence steps and timescales. (1) contact between two bubbles; (2) bubble interconnection during which bubble walls thin (bubbles assume an “eight” shape with cusps on their walls); (3) bubble coalescence after the rupture of the thinned films (replacement of the cusps in the “eight” shape by smooth bumps on the walls of the new coalesced bubble); (4) bubbles assume an oval shape and lastly recover to a spherical one.

**Fig. 4.**
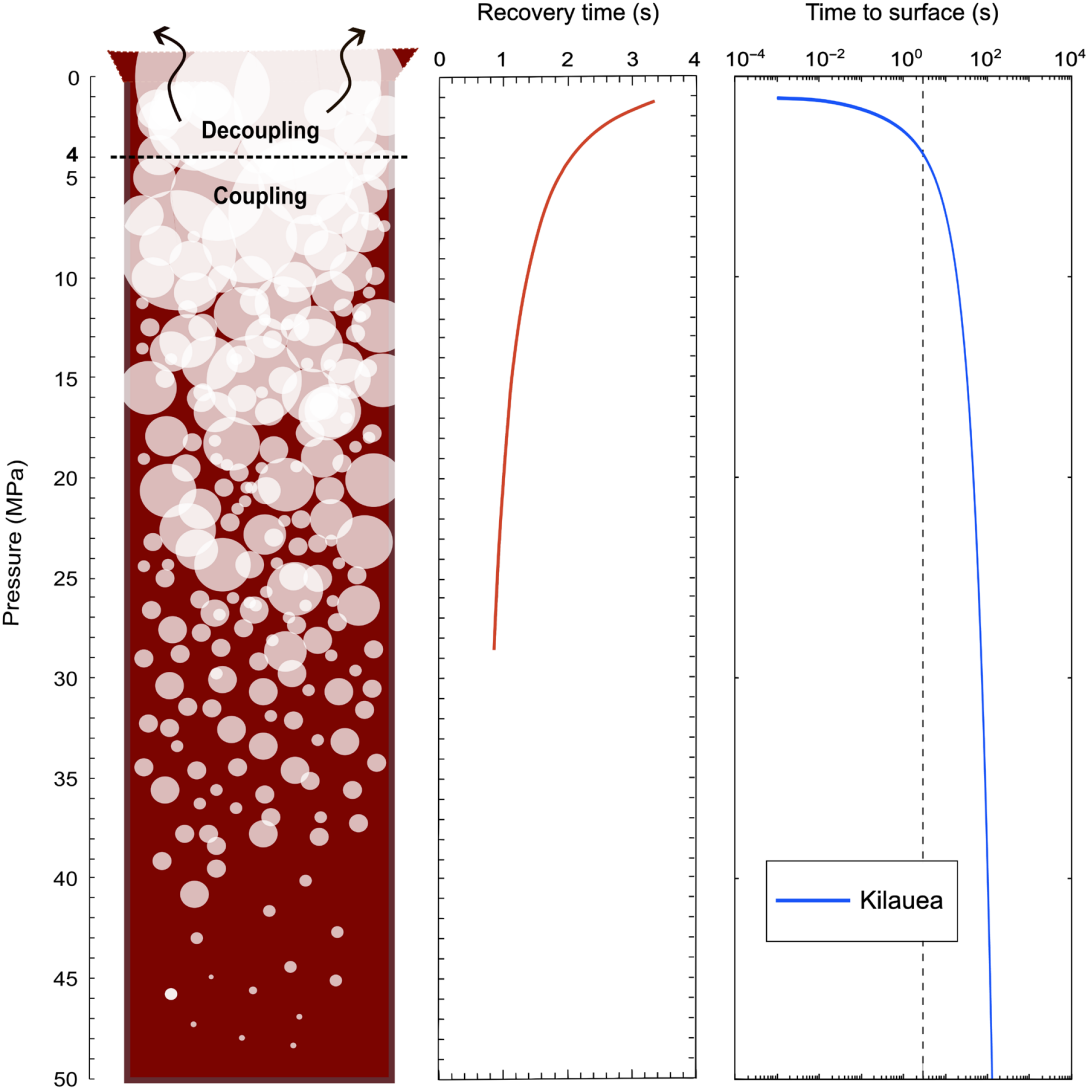
Sketch reporting bubble growth and coalescence within the conduit and mechanisms of coupling (up to the last ~100 m) and decoupling between volatiles and magma. Plots show bubble recovery time and magma time to surface versus pressure. In particular, the time required by magma to reach the surface for each pressure within the conduit using the lava fountaining simulations performed by La Spina *et al.* (*69*) for Kilauea is reported.

In the Superliq_Dec runs, it was possible to observe in real time and identify several steps and related times leading to bubble coalescence (table S7 and Fig. 3) including the following: (1) time of contact, when two separate bubbles enter in contact; (2) time of interconnection, when two bubbles are interconnected and their films start to thin; (3) time of coalescence, when there is an open connection between the bubbles as a result of the rupture of the thinned films; and (4) time of recovery, when the coalesced bubbles recover to a spherical shape. All of these steps can be observed in detail in Fig. 3. A similar sequence for bubble coalescence has been also observed by Masotta *et al.* (*58*) during in situ high-temperature observations of bubble growth in a rhyodacitic melt and by Ohashi *et al.* (*27*) during in situ decompression experiments of viscous Newtonian analogues in a mini-desiccator box. Some textural features of bubble coalescence [i.e., bubble-melt wall thinning by bending, stretching, and dimpling (*7*), corresponding to steps 1 and 2 of this study] have been observed in previous ex situ experimental decompression/vesiculation studies on both basaltic (*49*, *50*) and rhyolitic (*16*) compositions. As shown in this study, once two bubbles enter in contact (step 1) there is an interconnection (step 2) during which bubble walls thin until the film ruptures. During interconnection bubbles assume an “eight” shape with cusps on their walls. Because the film thickness is too thin to be resolved by radiography, we cannot see the rupture of the films in real time, but we can ascertain that it has occurred when we observe the replacement of the cusps in the “eight” shape by smooth bumps on the walls of the new coalesced bubble, and so bubbles coalescence (step 3). Once coalesced, bubbles assume an oval shape and lastly recover to a spherical one (step 4). The recovery time (i.e., the time required by coalesced bubbles to recover a spherical shape) is in the order of 1 to 3 s and results to be affected by pressure. We noted, indeed, an increase of the recovery time with decreasing pressure (Fig. 4). The recovery time (τ) for a coalesced pair of bubbles to return to a spherical shape can be compared with theoretical estimations (*7*, *21*, *32*, *72*–*74*). These allow us to predict τ from the rheological properties of the liquid, because retraction of the common wall between two interconnected bubbles is fostered by surface tension (σ) and resisted by the effective viscosity of the liquid (μ) ([τ = (*R*μ)/σ]). Theoretical results (table S7) were obtained assuming σ = 0.1 N m^−1^ (*7*, *73*), μ = 148 Pa·s [table S3; calculated using the model of Giordano *et al.* (*75*)], and *R* as the equivalent bubble radius (i.e., the radius of an undeformed sphere of equal volume; table S7). From the comparison, we noticed that the theoretical recovery times are faster than the experimental ones with a difference up to one order of magnitude at the lowest pressures (*P* < 3 MPa). However, fully understanding this discrepancy requires targeted and in-depth studies that are beyond the aim of this study.

Coalescence and degassing were also observed in the Subliq_Dec runs (movies S1C and S3). Evidence of coalescence events can be also found in the recovered samples. We can presume that large bubbles in backscattered electron (BSE) images (fig. S4, D to G) are the product of coalescence between two or more smaller bubbles. The presence of these coalesced bubbles implies that, at the end of the decompression in the Subliq_Dec runs, the formation of single large bubbles moving toward the top of the apparatus did not occur as in the Superliq_Dec experiments. This suggests that the formation of permeable pathways in the Superliq_Dec experiments allowed gas to escape, which can be related to the presence of microlite crystals. The role of microlite crystals on coalescence and formation of permeable pathways is still a debated topic. On the one hand, the presence of microlite crystals could lead to the following: (i) an increase in melt viscosity, as their presence indicates an increased silica content of the melt; (ii) an increase in bulk viscosity, which reduces film drainage ability and consequently the rate of coalescence (*13*); and (iii) a physical block or impediment of bubble expansion, movement, or coalescence. On the other hand, however, some studies (*76*–*78*) observed higher permeabilities and thus a more efficient degassing in crystal-bearing than in crystal-free basaltic magmas. This would suggest that the presence of crystals, forcing the bubbles concentration in some regions of the melt rather than in others, would facilitate the contact between the bubbles and therefore the formation of permeable pathways. The presence of crystals, slowing down the recovery time because of a higher viscosity, would favor a longer opening of permeable pathways and therefore a more efficient degassing.

### Influence of bubble coalescence on degassing

Volcanoes such as Etna, Stromboli, and Kilauea are generally characterized by the ascent of basaltic magma that involves different degrees of decoupled, open-system degassing, in which volatiles are able to flow faster than their originating, slowly ascending melt (*69*, *79*–*81*). In the presence of more viscous melts (such as rhyolitic melts), bubbles are relatively immobile with respect to the melt (i.e., the slip velocity of bubbles is negligible compared to magma ascent velocity), and this is commonly referred to as coupled or closed-system degassing (*82*, *83*), although in rhyolites open pathways may form, which allow degassing to take place (*84*, *85*). The style of basaltic explosive behavior is strongly related to the ability of gas to decouple from the melt, which depends on the relative rates of ascent of melt and bubbles, establishment of percolation bubble frameworks, and the extent of bubble coalescence (*80*, *82*, *86*, *87*).

While slow ascent rates, allowing gas bubbles to decouple from the melt, move, and coalesce, increase the likelihood of a Strombolian or effusive eruption (*79*, *88*), fast ascent rates such as estimated for basaltic Plinian and sub-Plinian eruptions [with average rates ranging between 4 and 75 m s^−1^; (*62*, *89*, *90*)] likely restrict the time available for gas-melt decoupling during magma ascent, promoting, depending on magma viscosity, fragmentation and an explosive eruption or high-intensity fountain activity (*66*, *68*, *69*, *80*, *91*–*94*).

In this study, we measured bubble recovery time in the Superliq_Dec runs and the ascent time of Kilauea magma to investigate the role of bubble connections on degassing [i.e., both closed-system degassing (volatile exsolution and vesiculation) and open-system degassing (outgassing); (*95*)] of low-viscosity crystal-poor/crystal-free basaltic magmas. The quicker this process, the more likely a permeable pathway closes before connecting with the vent of the conduit, preventing gas from decoupling efficiently from the melt. For basaltic lava fountaining activity on Kilauea, La Spina *et al.* (*69*) show a maximum ascent velocity of ~60 m s^−1^ [average velocity ~ 15 m s^−1^ consistent with Ferguson *et al.* (*96*)], which means that magma would need more than 3 s to reach the surface from a depth greater than 200 m. Our obtained recovery timescales are on the order of ~3 s at low magmatic pressures, but they are even shorter at higher pressures, implying that the recovery of spherical shape at depth may occur too quickly to allow permeable pathways consisting of chains of interconnected bubbles to reach the surface and connect with the vent during fountaining. To better show this, we used results from the lava fountaining simulations performed by La Spina *et al.* (*69*) for Kilauea, and we used their model to extrapolate the time required by magma to reach the surface for each pressure. From Fig. 4 and table S8, we note that the time to reach the surface becomes greater than 3 s when the pressure is greater than ~4 MPa (~90-m depth). This implies that, for pressures higher than ~4 MPa, the formation of permeable pathways is mostly inhibited, and thus, the gas-melt system remains coupled at least up to the last 100 m of the conduit (Fig. 4). In general, our results show that lava fountaining eruptions at low-viscosity basaltic volcanoes (such as at Kilauea) are associated with rapid bubble coalescence and recovery time and a high magma ascent rate, resulting in a coupled behavior until the last ~100 m of the conduit (*69*, *79*, *80*). We speculate that some degree of decoupling may occur in the shallowest part of the conduit; otherwise, it is likely that intense fragmentation would be produced by closed-system degassing. This has been observed, for instance, in basaltic pumices from the paroxysmal events of Stromboli where bubble populations are consistent with closed-system degassing. Close to the surface, where the gas volume fraction is high, the occurrence of multiple events of coalescence at the same time may generate permeable pathways allowing some degree of open-system degassing. Such a process may contribute to near-surface open-system degassing in slow-ascending magmas, such as within a lava lake.

Another possible mechanism for gas-magma decoupling that might happen during ascent is the formation of a slug flow due to the coalescence of numerous smaller bubbles (*97*, *98*). Our Superliq_Dec experiments show that, at low pressures, the increase in coalescence events produce ultimately a very large bubble, which indicates that the formation of a slug flow in the shallowest part of the conduit is possible. A high gas volume fraction (>0.6) (*99*) is required to reach the size of the conduit and develop a slug flow. For a low–volatile content magma, such as that of Kilauea, this high gas volume fraction is obtained in the shallowest part of the conduit, where most of the exsolution occurs. The formation of slug flow may occur also at depth due to accumulation of gas entrapped at some geometrical discontinuities within the plumbing system (*97*), but this would generate Strombolian, rather than lava fountain, activity (*81*, *97*, *98*).

Although in the Subliq_Dec runs we cannot visualize in detail the steps that lead to bubble coalescence through radiography, we can, however, obtain some insights on magma-gas coupling/decoupling. We observe that, in the presence of a more viscous magma (η ≥ 10^3^ Pa·s), in case of a rupture of the film between bubbles, the recovery time is much longer than that obtained for the less viscous Superliq_Dec runs. This longer recovery time promotes the formation of degassing pathways that allow gas to escape. An increase in magma viscosity, indeed, can affect the eruptive behavior in several ways such as increasing the fragmentation capability due to bubble overpressure (*100*, *101*), suppressing large bubble floatation, and increasing the capillary number and the role of shear deformation (*102*, *103*). Our novel experiments with an in situ view show a slow bubble expansion rate and consequently a long recovery timescale at subliquidus conditions (movies S1C and S3), which supports assumptions of previous studies (*72*, *77*, *104*, *105*). This result suggests that high viscosity produced by microlite crystallization restricts bubble growth and expansion and extends bubble coalescence time, promoting connected pathways between bubbles (as those visible in movie S3 from minute 15 onwards) and thus increased connectivity, which, in turn, favor outgassing (*72*, *77*, *95*, *106*, *107*).

Our work represents a substantial step forward in the understanding of magma and gas dynamics, even though it has limitations that result from the use of 2D radiography and inability to apply a shear stress (which are likely to affect bubble growth and coalescence). Thanks to the new IHPV apparatus presented here, we were able to capture and study the vesiculation kinetics in basaltic magmas in real time, in situ, and at pressures and temperatures compatible with those of basaltic volcanoes. Our novel x-ray transparent apparatus has proved to be an invaluable tool to capture and quantify kinetic of bubble formation (i.e., bubble growth, expansion, and coalescence) and magma dynamics (i.e., degassing and gas-magma coupling/decoupling) at syneruptive conditions. In this regard, the growth rates derived from our experiments represent a noteworthy contribution, as they confirm estimations calculated using numerical and theoretical models.

Future developments of the x-ray transparent IHPV will be dedicated to allowing fast synchrotron x-ray tomography of magmatic samples at high-pressure and high-temperature conditions, to visualize and quantify the vesiculation process (nucleation, growth, and coalescence) directly in 4D (3D space plus time) both at superliquidus and subliquidus conditions. This will allow us to further improve first the current numerical model by integrating previously unknown constraints, and then our understanding of magma behavior at pre- and syneruptive conditions and the related volcanic hazard.

## MATERIALS AND METHODS

### Starting material

The starting material used for our vesiculation experiments is a trachybasalt from the lower vents of the 2001 Mt. Etna eruption (*62*, *108*–*111*). We used hydrous, crystal-free basaltic samples from Etna with different water contents (0.5 to 2 wt %) and a rhyolitic sample with 0.2 wt % of water. The anhydrous glassy starting material (table S1) was synthesized by melting a crushed rock sample in a Pt crucible. Melting was performed in a Nabertherm MoSi_2_ box furnace at 1400°C and at atmospheric pressure. The melt was left in the furnace for 4 hours to fully degas and dissolve any crystals present. The melt was then quenched in air to glass, and this procedure was repeated twice to enhance homogenization. Hydrous starting glasses with 0.5, 1, and 2 wt % H_2_O were obtained by melting the starting material and homogenizing it with H_2_O in Au_80_Pd_20_ capsules at 100 MPa and 1200°C using a Titanium Zirconium Molybdenum (TZM) cold-seal pressure vessel apparatus at the School of Earth Sciences, University of Bristol, UK. The water content of the starting materials was confirmed to be present in the glasses by Fourier Transform Infrared spectroscopy. The FTIR measurements were performed in transmission mode by a PerkinElmer Spotlight 400 spectrometer equipped with a Mercury Cadmium Telluride (MCT; or HgCdTe) array detector cooled with liquid N_2_ at the Department of Earth and Environmental Sciences at the University of Manchester, UK. Spectra were collected by accumulating 64 scans using a square aperture of 100 μm across with a spectrum resolution of 4 cm^−1^. Spectra were analyzed using Spectragryph (*112*). Using the density trend and Etna basalt extinction coefficient of Testemale *et al.* (*113*), the molecular H_2_O peak at 3550 cm^−1^ gives 2.05 ± 0.01 wt % for Etna 4, 1.32 ± 0.01 wt % for Etna 12, 1.48 ± 0.01 wt % for Etna 13 and 1.13 ± 0.01 wt % for Etna 15 starting materials.

### In situ high-pressure, high-temperature synchrotron x-ray radiography experimental apparatus

In situ high-pressure, high-temperature experiments were performed at the x-ray tomography/radiography beamline I12-JEEP, Diamond Light Source, Harwell, UK. We used a dedicated x-ray transparent IHPV apparatus developed at Neel Institute and based on a previous one (*113*) combined with x-ray radiography to perform in situ vesiculation experiments under water-saturated conditions at crustal pressures. The IHPV apparatus was pressurized with He, which allowed us to precisely control the decompression rate during in situ experiments and to quantify disequilibrium in basaltic magmas after pressure perturbations. The pressurization was controlled by a pressure regulator (*114*). The vessel is characterized by the placement of the furnace inside the vessel (internally heated). The vessel is a thick-walled steel cylinder having both ends open. The open ends are closed by heads through which pressure, electrical lead, and thermocouple lead enter. The vessel has two sapphire windows at 180°, which allow the x-ray beam to enter the vessel, passing through the sample and reaching the camera for radiography acquisitions. Temperature was measured with a K-type thermocouple positioned close to the sample in the middle of the furnace hotspot. The K-type thermocouple measures the sample temperature with an uncertainty of ±0.5°C. The sample holder was an alumina, which is suitable for the temperature range investigated and has a low x-ray attenuation coefficient. The hydrous glass (~1.5 mm by 3 mm by 5 mm; ~22 mm^3^ once melted) was placed in the customized alumina crucible whose sizes are reported in fig. S1.

### Experimental strategy

We combined fast x-ray synchrotron radiography with our novel IHPV apparatus to quantify bubble growth and coalescence in basaltic magmas during decompression. The experiments focused on bubble kinetics as a function of initial pressure, decompression rate, and H_2_O content. In all the experiments, we placed an Etna basalt with approximately 0.5 to 2 wt % of water in the sample crucible, except for the one in which we used a rhyolitic sample. We pressurized the system at first with gas (He), and then we heated up to 1180°C with a heating rate of 0.75°C s^−1^. At this point, we continued the experiments by keeping isothermal conditions (1180°C; Superliq_Dec experiments; table S2) or dropping the temperature to different target isothermal conditions (1050° to 1080°C; Subliq_Dec experiments; table S2) with a cooling rate of 0.75°C s^−1^. After that, we dropped the pressure to 0.1 MPa with a decompression rate between 0.03 and 0.08 MPa s^−1^ to simulate different ascent rates during basaltic eruptions, starting decompression at different initial pressures (75, 50, 30, and 20 MPa; table S2). Once reached 0.1 MPa, the temperature was dropped to the ambient one with a cooling rate of 0.75°C s^−1^ (fig. S2).

### In situ synchrotron x-ray radiography acquisition

The x-ray radiography beamline I12-JEEP (Diamond Light Source, Harwell, UK) allowed us to perform experiments using monochromatic 53-keV x-rays, a pixel size of 6.642 μm, and a scanning time of 40 ms per frame to achieve 25 frames per second, at a sample to detector distance of 35 cm, an exposure time for a single projection of 15 ms, and an acquisition time of 988.28 s, for a total of 24,707 images. The acquisition of radiographic projections began shortly before the start of decompression and covered the entire decompression path until ambient pressure was reached.

### Image processing and analysis

The radiographic images were processed and stacked using ImageJ software (*115*) to obtain movies (movie S1). Movies were made by importing in ImageJ the radiographic images in TIFF format as image sequences and then saved as an AVI file. The movies reported in movies S1 to S3 were edited by using DaVinci Resolve (version 18.0.4) video editing software. ImageJ was also used to measure bubble diameter and area. First of all, for all the runs, we used “set scale” to convert pixel in micrometers (1 pixel = 6.642 μm); then frames were converted in 8-bit and then adjusted by brightness/contrast to better highlight bubbles from melts. Because of the low contrast between bubbles and melt and to bubble overlays, it was not possible to use the “threshold” tool and the “tracking plugin.” For the Superliq_Dec runs bubbles diameter and area were measured by manually tracking bubbles using “oval selections” and then the “measure” tool. To better highlight edges of the bubbles the “Find edges” tool was also applied. For the Subliq_Dec runs, instead, because of the absence of spherical bubbles, bubble area was measured manually by contouring bubble edges using the “polygon selections” and then the measure tool.

### Scanning electron microscope and electron microprobe analysis

BSE images were collected using a FEI Quanta 650 FEG-SEM electron microscope in the Department of Earth and Environmental Sciences, University of Manchester, UK, to analyze vesicles shapes and crystals morphologies. We used an acceleration voltage of 15 kV and a working distance of 10 mm. The starting material (glass) and the samples obtained during in situ vesiculation experiments were analyzed with a JEOL JXA-8530F field-emission electron microprobe at the Photon Science Institute, University of Manchester, UK. The operating conditions were as follows: 15-kV accelerating voltage, 10-nA beam current, and a beam diameter of 10 or 5 μm. Na and K were measured first to minimize loss by volatilization. Calibration standards were albite for Na, periclase for Mg, corundum for Al, fayalite for Fe, tephroite for Mn, apatite for P, sanidine for K, wollastonite for Ca and Si, and rutile for Ti.

## Supplementary Materials

### This PDF file includes:

Supplementary Text Figs. S1 to S7 Legends for tables S1 to S8 Legends for movies S1 to S3 References
