# Supplementary material for: Direct observation of degassing during decompression of basaltic magma
Source: Sci Adv. 2024 Aug 16;10(33):eado2585. doi: 10.1126/sciadv.ado2585 (PMC11421694; doi:10.1126/sciadv.ado2585)
Supplement: Supplementary file 1 — Supplementary Text Figs. S1 to S7 Legends for tables S1 to S8 Legends for movies S1 to S3 References [file sciadv.ado2585_sm.pdf]

Science Advances
AAAS

Supplementary Materials for

Direct observation of degassing during decompression of basaltic magma

Barbara Bonechi *et al.*

Corresponding author: Barbara Bonechi, barbara.bonechi@manchester.ac.uk

Sci. Adv. **10**, eado2585 (2024)
DOI: 10.1126/sciadv.ado2585

The PDF file includes:

Supplementary Text
Figs. S1 to S7
Legends for tables S1 to S8
Legends for movies S1 to S3
References

Other Supplementary Material for this manuscript includes the following:

Tables S1 to S8
Movies S1 to S3

Supplementary Text

Evidence of different vesicle and crystal textures in the recovered samples

BSE images of recovered Superliq_Dec runs show portions of melt at the edges of the crucible while the inner part was extruded during the experiments (Fig. S4a). These portions are characterized by the presence of vesicles (i.e., the equivalent of bubbles in the recovered, solidified products) with spherical shape and crystals with disequilibrium texture. These latter formed during the final continuous cooling path (Fig. S2), as previously observed in the work of Arzilli et al. *(62)*. The phase assemblage consists of clinopyroxene (cpx), olivine (ol), plagioclase (plg), oxide (ox) and glass. Cpx (of augitic/diopsidic composition; $En_{35–37}Fs_{15–21}Wo_{43–48}$; Fig. S5a) shows dendritic morphology with very small sizes up to 5-10 μm (Fig. S4b). Ol (Fo_{86-87}) is characterized by growth textures while plg (of bytownitic composition; $Ab_{69-70}Or_{28-29}An_2$; Fig. S5b) shows swallow-tailed morphology and is crystallized at the edges of the crucible (Fig. S4b). Ox shows more euhedral textures with small sizes up to 10 μm. Among this group, the Rhyo sample is completely glassy with no crystals due to the high temperature (1180 °C; Fig. S6a) above its liquidus ($T_{liquidus}$ ~1100 °C). The texture is typical of a polyhedral foam *(116)*, with tightly packed, polyhedral bubbles separated by planar glass films meeting in threes along edges called plateau borders *(117)* (Fig. S6b). Differently from the Superliq_Dec runs, where a large part of the melt was extruded during the experiments, in the runs of the Subliq_Dec group there is evidence of the presence of bubbles since the latter remained trapped in the melt and could not escape due to the higher viscosity. BSE images of recovered Subliq_Dec samples, indeed, show numerous vesicles with tortuous outlines often as a result of several bubbles coalesced together (Fig. S4e-g). Among these runs it is possible to note a variation of vesicle size and amount as function of viscosity and water content (Table S3). Indeed, the less viscous and more hydrated run (i.e., Etna 4_1; Fig. S4e) is characterized by numerous vesicles of large size (up to 1 mm), while the most viscous and less hydrated run (i.e., Etna 3; Fig. S4g) shows fewer vesicles of small size (up to 0.5 mm). In addition, in the latter run there are portions more deformed with small vesicles and others less deformed with larger vesicles. Usually in the most deformed portions crystals show very small sizes (<10 μm), while around the large vesicles they are larger (up to 30 μm; Fig. S4d). The phase assemblage and composition is the same as the other group (i.e., cpx+ol+plg+ox±glass). Differently from the other group, crystals show more euhedral shapes (Fig. S4d-h) and this could be related to the fact that crystallization occurred before the decompression path as a consequence of the single step cooling. Ox are very tiny (up to 2 μm) and in great amount often at the rims of vesicles and crystals or overlapping cpx crystals (Fig. S4h).

Bubble expansion rate

Since in the Subliq_Dec runs and in the Rhyo sample bubbles are not spherical, it is not possible to extrapolate the third dimension from the radiography images and we cannot retrieve the growth rate from the calculated expansion rate. Results are reported in Tables S3 and S6. We note that the average expansion rate (E_R) is directly proportional to temperature (except for Rhyo sample) and water content (Fig. S7a-b), while it is inversely correlated with crystal volume fraction and viscosity (Fig. S7c-e). Crystal volume fraction (ϕ) and viscosity (η), which we cannot directly measure during the experiments, are

related to experimental conditions after the single step cooling and before the start of the decompression path and are calculated using the software Rhyolite-MELTS *(118, 119)* and the model of Giordano et al. *(75)* and Vona et al. *(120)*. If we consider the variation of the expansion rate with pressure, we can observe that bubble expansion increases with decreasing pressure (Fig. 2a). Another interesting aspect is that bubble expansion starts later in time (at lower P) for higher viscosity samples (Fig. 2a), which promotes higher strain and lower H_2O diffusion, and thus makes it difficult for bubbles to expand. This time delay, that we observed here for the first time in real time, was previously observed by Proussevitch et al. *(31)*, Sparks *(1)*, Proussevitch and Sahagian *(45)*, Liu and Zhang *(55)*, Lyakhovsky et al. *(56)*, Burgisser and Gardner *(121)*, Huber et al. *(25)*, Lensky et al. *(51)*. According to these authors the time delay represents "the interval between nucleation and the time the bubble starts to grow in accordance with the parabolic law" and it is function of the initial bubble size and of a dynamic factor depending on volatile oversaturation and melt properties (e.g., viscosity, diffusivity). Furthermore, we also observed that the bubble area increases rapidly with decreasing P in the less viscous runs. This is because the expansion velocity is controlled also by the viscosity of the liquid surrounding the bubbles. For comparison, we calculated bubble ΔE_R also in the Superliq_Dec runs (Tables S3 and S6). Since in these runs it was not possible to discern between coalescing or non-coalescing bubbles, in order to have comparable data, the ΔE_R for the Superliq_Dec runs was calculated for both bubbles that coalesce and do not coalesce. We noted that the ΔE_R increases with decreasing pressure as it also does for the other group (Fig. 2b). Moreover, the expansion is more rapid for bubbles nucleated at the end of the experiment (Fig. 2b) due to the overpressure of bubbles compared to the pressure of the melt, and to the oversaturation of volatiles as previously observed for the growth rates. Finally, considering the basaltic samples, it is interesting to note that the maximum expansion rate for the Superliq_Dec (ΔE_R = ~10^4 and ~10^6 $\mu m^2 \cdot s^{-1}$ for uncoalesced and coalesced bubbles, respectively) and Subliq_Dec (ΔE_R = ~10^3 $\mu m^2 \cdot s^{-1}$) runs varies of ~1-3 orders of magnitude. This can be related to their different viscosity before the decompression path. Indeed, according to Rhyolite-MELTS and Giordano et al. *(75)* modelling the Superliq_Dec runs are less viscous (log η = ~2 Pa·s; ϕ = 0 %) than those of the other group (log η = ~3-4 Pa·s; ϕ = 8-45 %) as consequence of the higher temperature (1180 °C) that is above the liquidus (Fig. S2) and prevents crystallization.

Supplementary Figures

Fig. S1.

a) Experimental apparatus, (b) alumina crucible and (c) Autoclave/Regulation-Pressure unit description parts. The vessel is characterized by the placement of the furnace inside the vessel (internally heated). The vessel is a thick-walled steel cylinder having both ends open. The open ends are closed by heads through which pressure, electrical and thermocouple lead enter. The vessel has 2 sapphire windows at 180°, which allow the X-ray beam to enter the vessel, passing through the sample, and reaching the camera for radiography acquisitions. This apparatus combined with fast synchrotron X-ray radiography (at beamline I12-JEEP, Diamond Light Source, Harwell, UK) allowed us to capture the vesiculation in real time.

a)

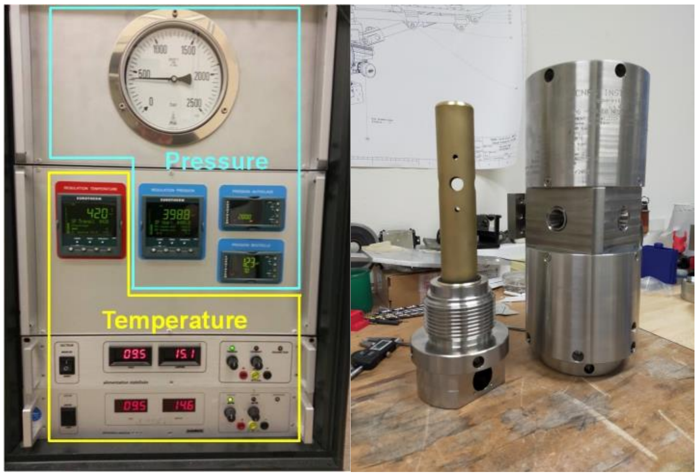

b)

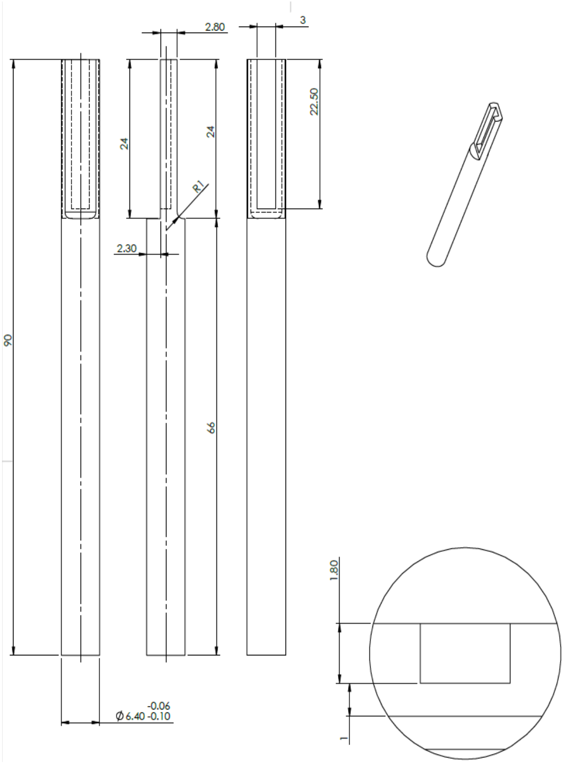

c)

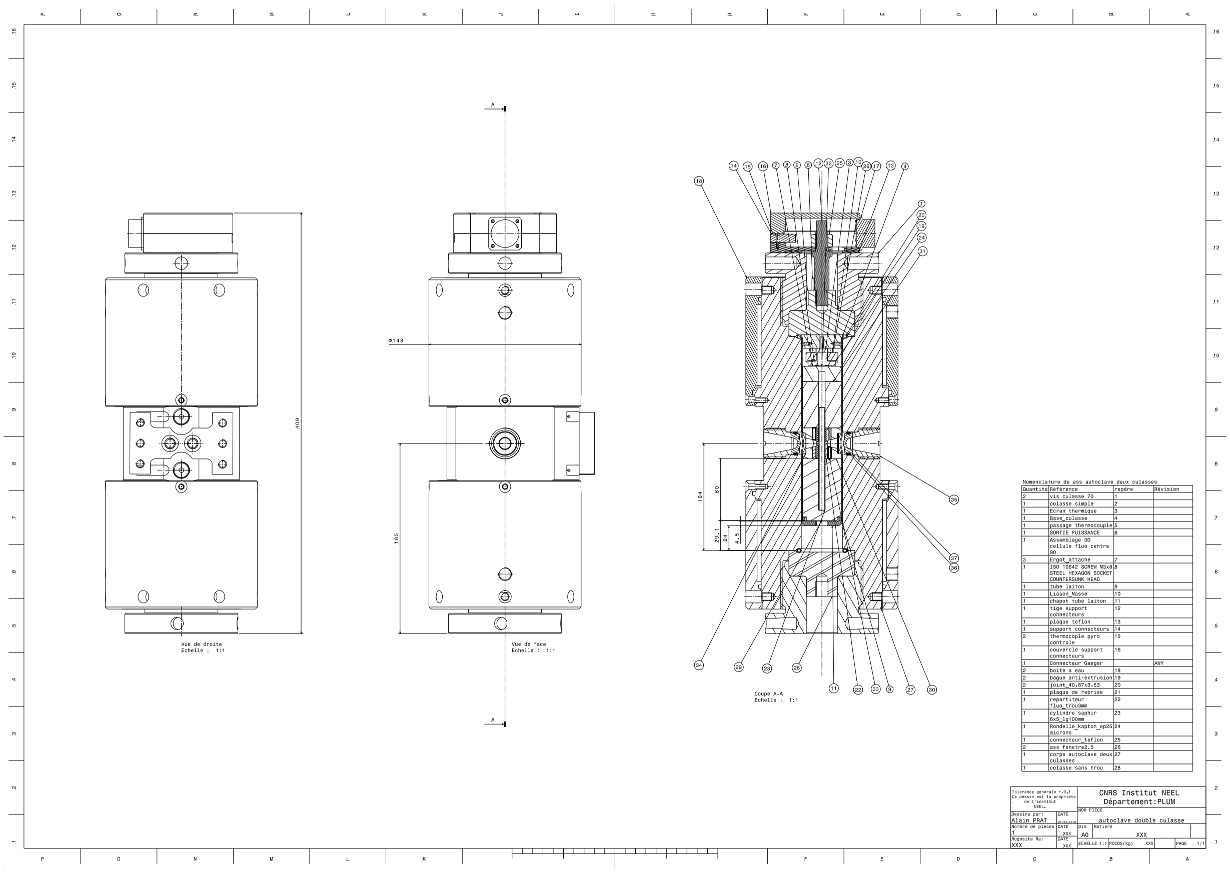

Quantité	Référence	repére	Révision
2	vis culasse 70	1	
1	culasse simple	2	
1	Ecran thermique	3	
1	Base_culasse	4	
1	passage thermocouple	5	
1	SORTIE PUISSANCE	6	
1	Assemblage 3D cellule fluo centre 90		
3	Ergot_attache	7	
1	ISO 10642 SCREW M3x8 STEEL HEXAGON SOCKET COUNTERSUNK HEAD	8	
1	tube laiton	9	
1	Liason_Masse	10	
1	chapot tube laiton	11	
1	tige support connecteurs	12	
1	plaque teflon	13	
1	support connecteurs	14	
2	thermocople pyro controle	15	
1	couvercle support connecteurs	16	
1	Connecteur Gaeger		ANY
2	boite a eau	18	
2	bague anti-extrusion	19	
2	joint_40.87x3.53	20	
1	plaque de reprise	21	
1	repartiteur fluo_trou3mm	22	
1	cylindre saphir 6x5_lg100mm	23	
1	Rondelle_kapton_ep25 microns	24	
1	connecteur_teflon	25	
2	ass fenetre2.5	26	
1	corps autoclave deux culasses	27	
1	culasse sans trou	28	

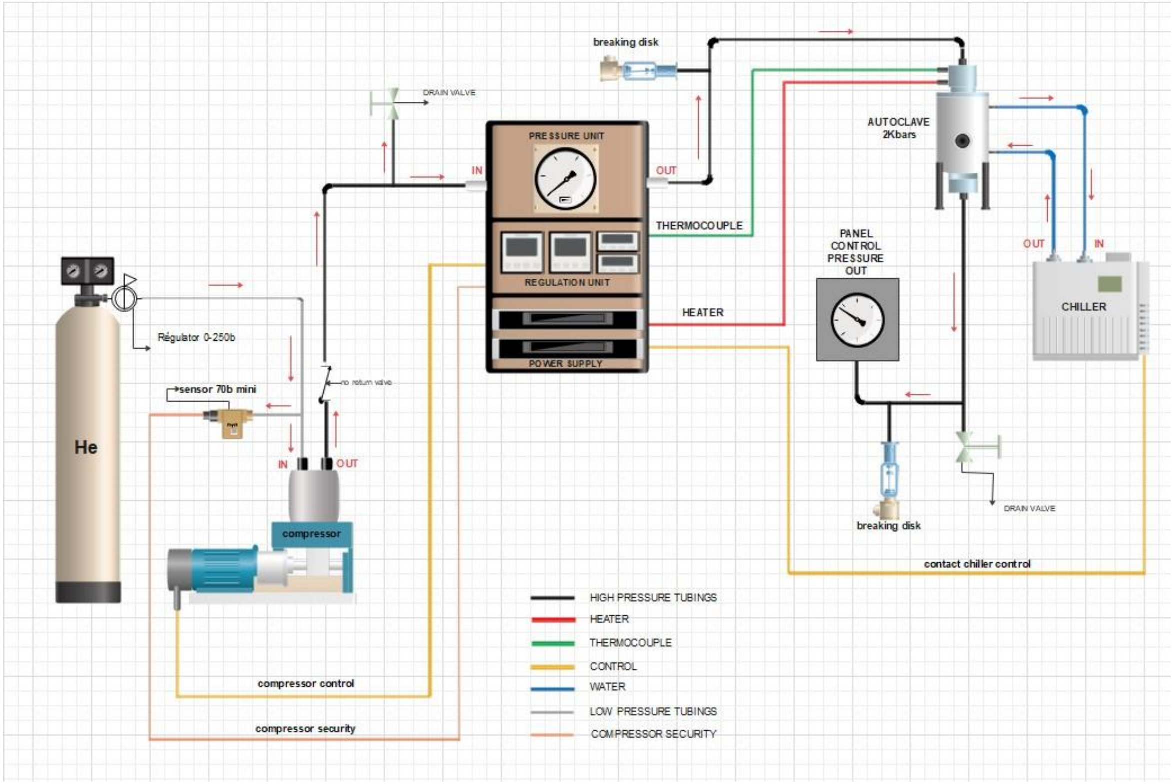

Fig. S2.

Experimental conditions. a, b Liquidus temperature ($T_{liquidus\ cpx}$) is obtained with Rhyolite-MELTS software, using the 2001 Etna trachybasaltic composition (Table S1). In all the experiments (a1-b3) the system was first pressurized at different pressure (75, 50, 30 and 20 MPa) and then heated up to 1180 °C with a heating rate of 0.75 $°C·s^{-1}$. Four experiments (Superliq_Dec; a1-a3) were performed keeping isothermal condition (1180 °C) while other five experiments (Subliq_Dec; b1-b3) by dropping the initial temperature (1180 °C) to different target isothermal conditions (1050-1080 °C) with a cooling rate of 0.75 $°C·s^{-1}$. After that in all the experiments the initial pressure was dropped to 0.1 MPa with a decompression rate between 0.03 and 0.08 $MPa·s^{-1}$. Once reached 0.1 MPa, the temperature was dropped to the ambient one with a continuous cooling rate of 0.75 $°C·s^{-1}$.

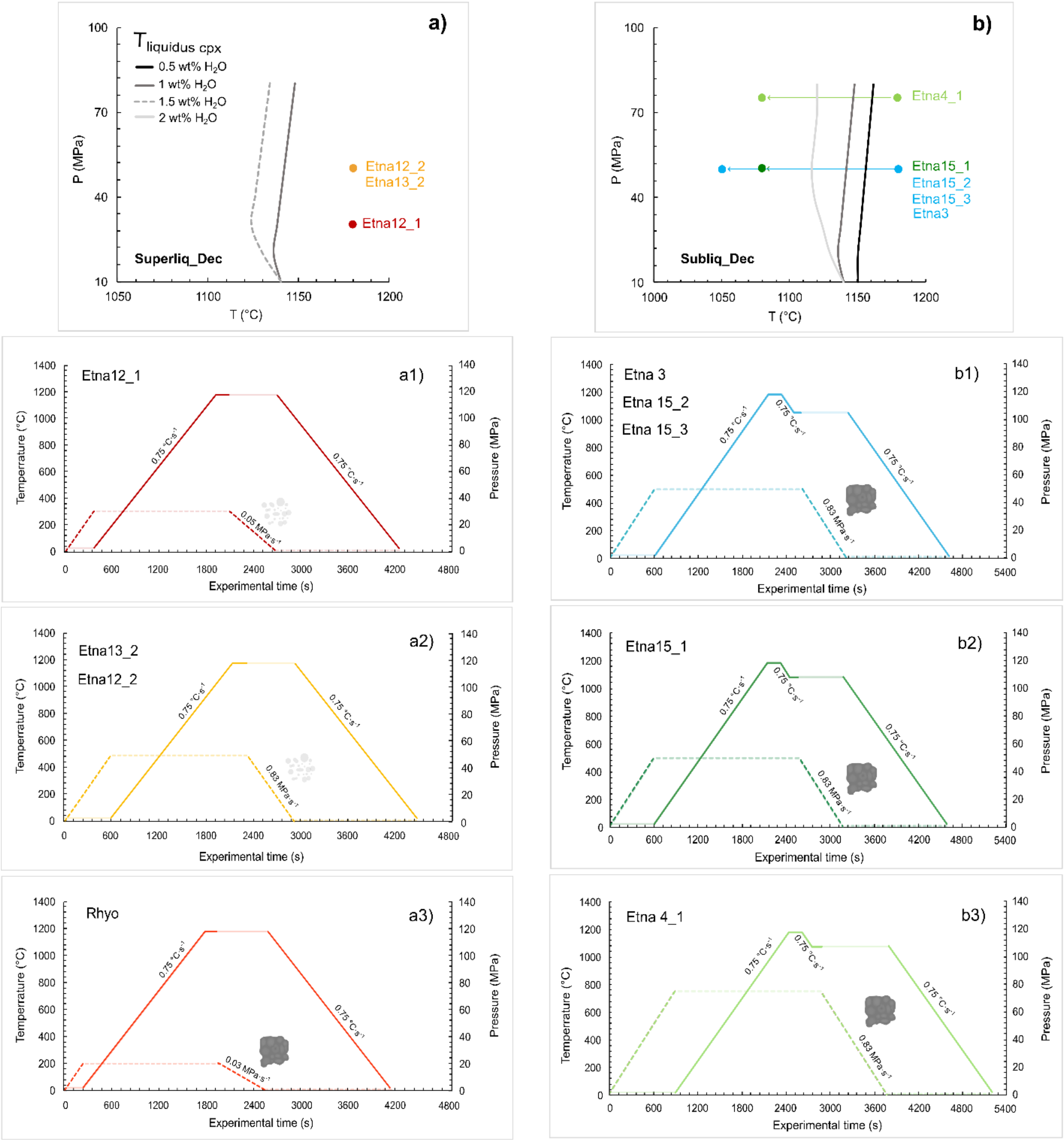

Fig. S3.

Diagrams showing incremental bubble growth rate vs pressure for the Superliq_Dec experiments. The bubble growth rates estimated from the decompression experiments were compared with those calculated using the numerical bubble growth model of Coumans et al. *(44)*. Numerical results (Table S5) are obtained assuming different decompression rates (0.05 and 0.08 MPa s^{-1}), different volatile contents (1 and 2 wt.% of H_2O) and different bubble number densities (N_b = 10^{10}-10^{13} m^{-3}). Plotted numerical simulations have been computed using the Etna composition (Table S1) and a magma density of 2700 kg m^{-3}. The decompression rates (MPa·s^{-1}) for the experimental run are reported in brackets.

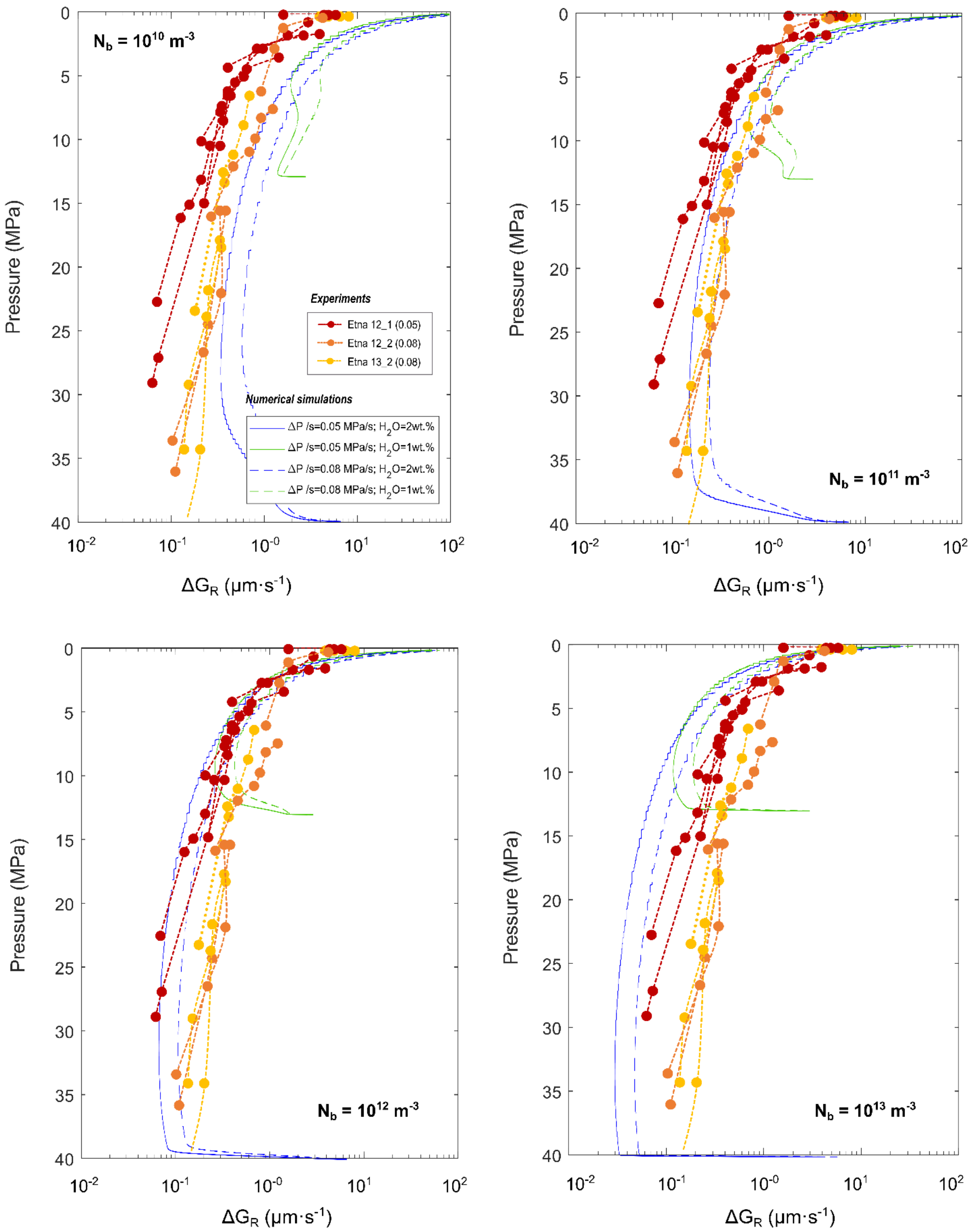

Fig. S4.

BSE images of recovered Superliq_Dec (a-c) and Subliq_Dec (d-h) runs. a) Image of Etna 13_2 run shows portions of melt at the edges of the crucible with the presence of spherical vesicles, while the inner part was extruded during the experiments. b-c) Images of Etna 12_1 run show crystals with disequilibrium texture formed during the final continuous cooling path. d-g) Images showing vesicles shape and size of Subliq_Dec runs. h) BSE image of Etna 3 run showing the great amount of very tiny oxides often present at the rims of bubbles.

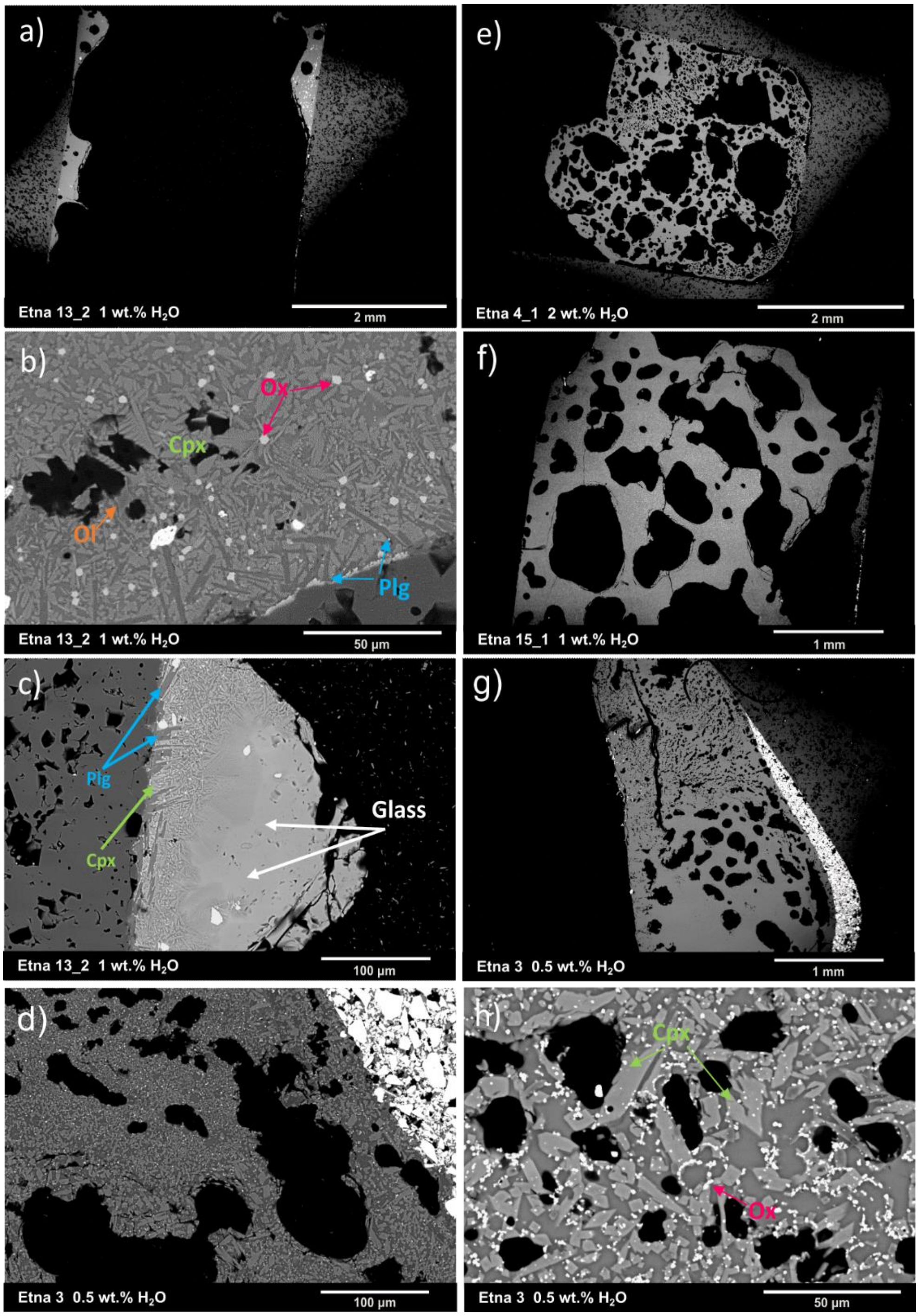

Fig. S5.

Chemical compositions of clinopyroxene (a) and feldspar (b) crystals. En: enstatite; Fs: ferrosilite; Di: diopside; Hd: hedenbergite; Wo: Wollastonite; An: Anorthite; Ab: Albite; Or: Orthoclase.

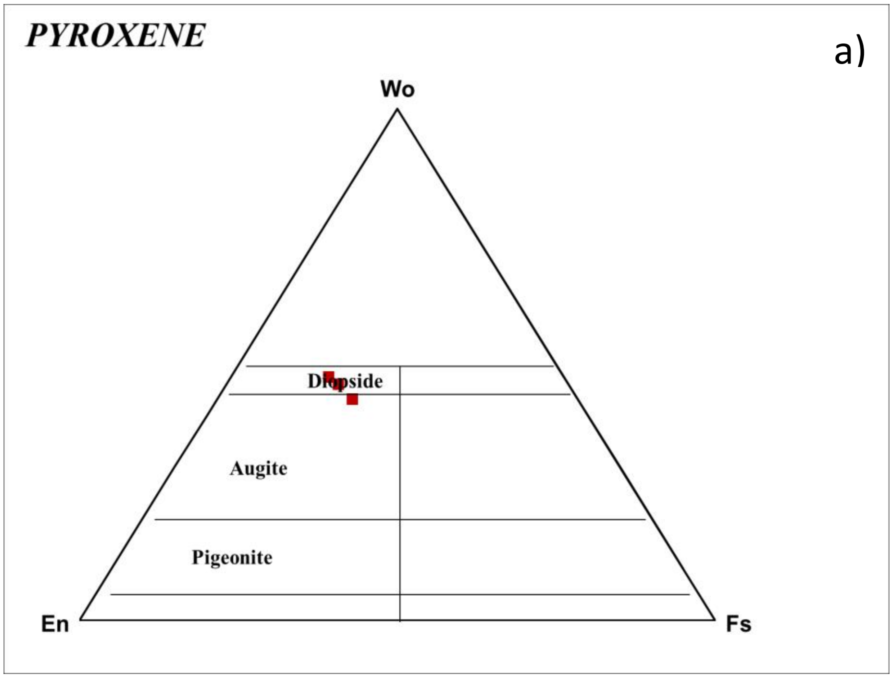

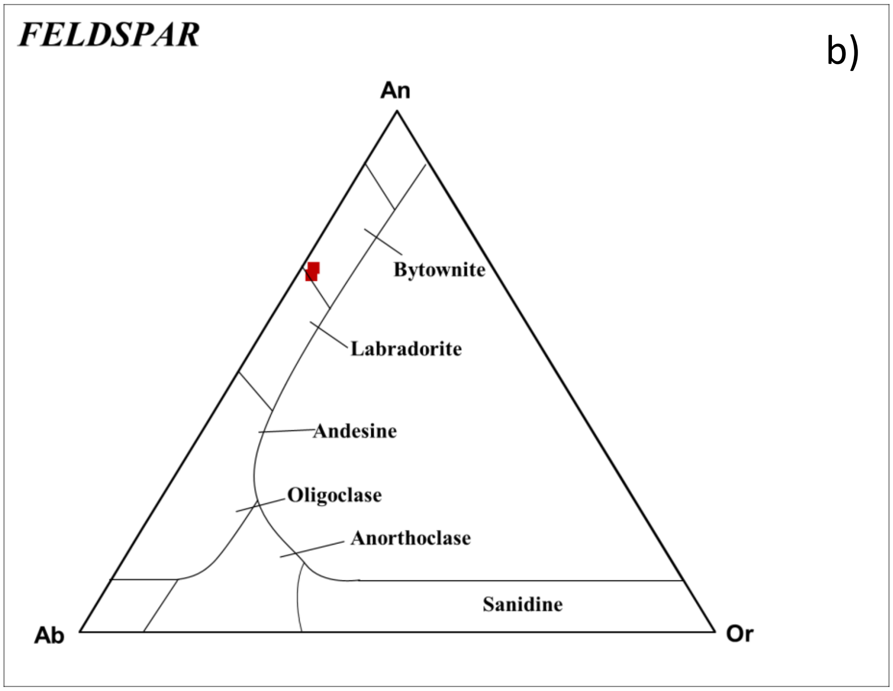

Fig. S6.

BSE images of Rhyo sample. a) Image shows that the sample is completely glassy with no crystals; b) image showing the polyhedral foamy texture.

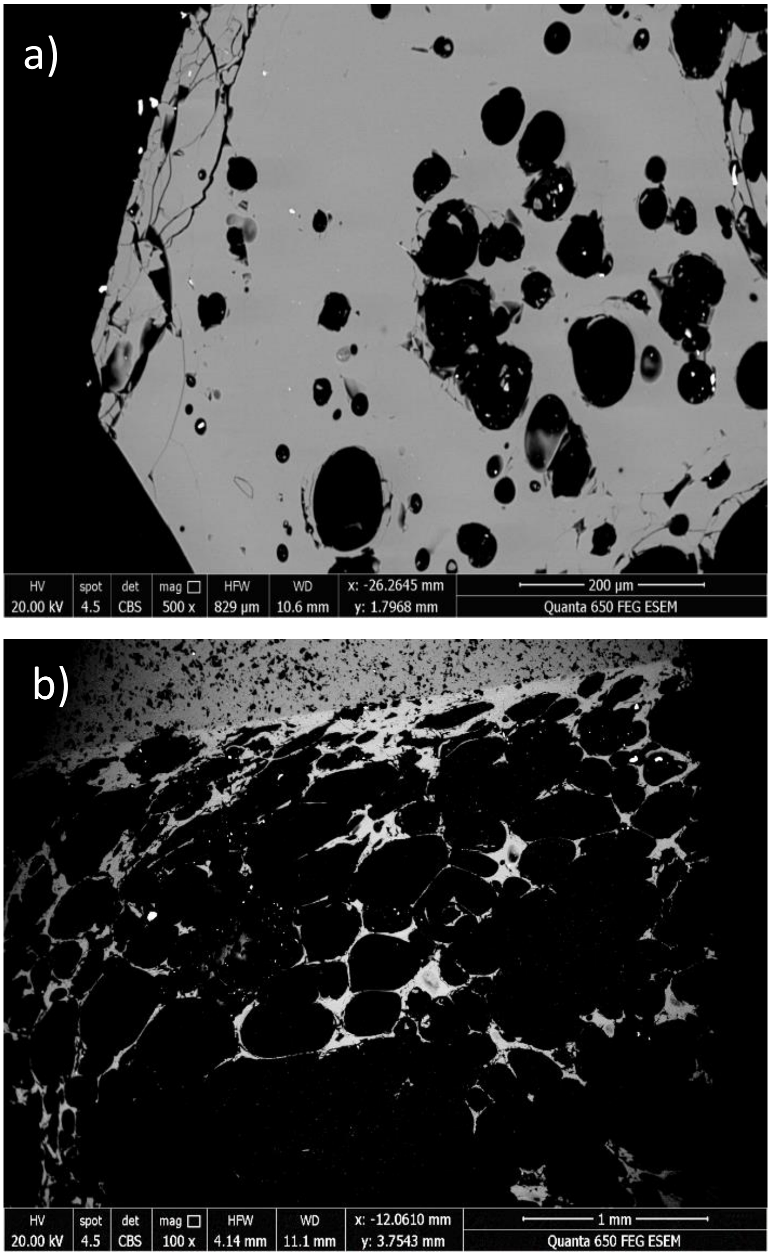

Fig. S7.

Diagrams showing average expansion rate vs a) temperature, b) H_2O (initial content), c) crystal volume fraction, d) melt viscosity and e) bulk viscosity for Subliq_Dec runs and Rhyo sample.

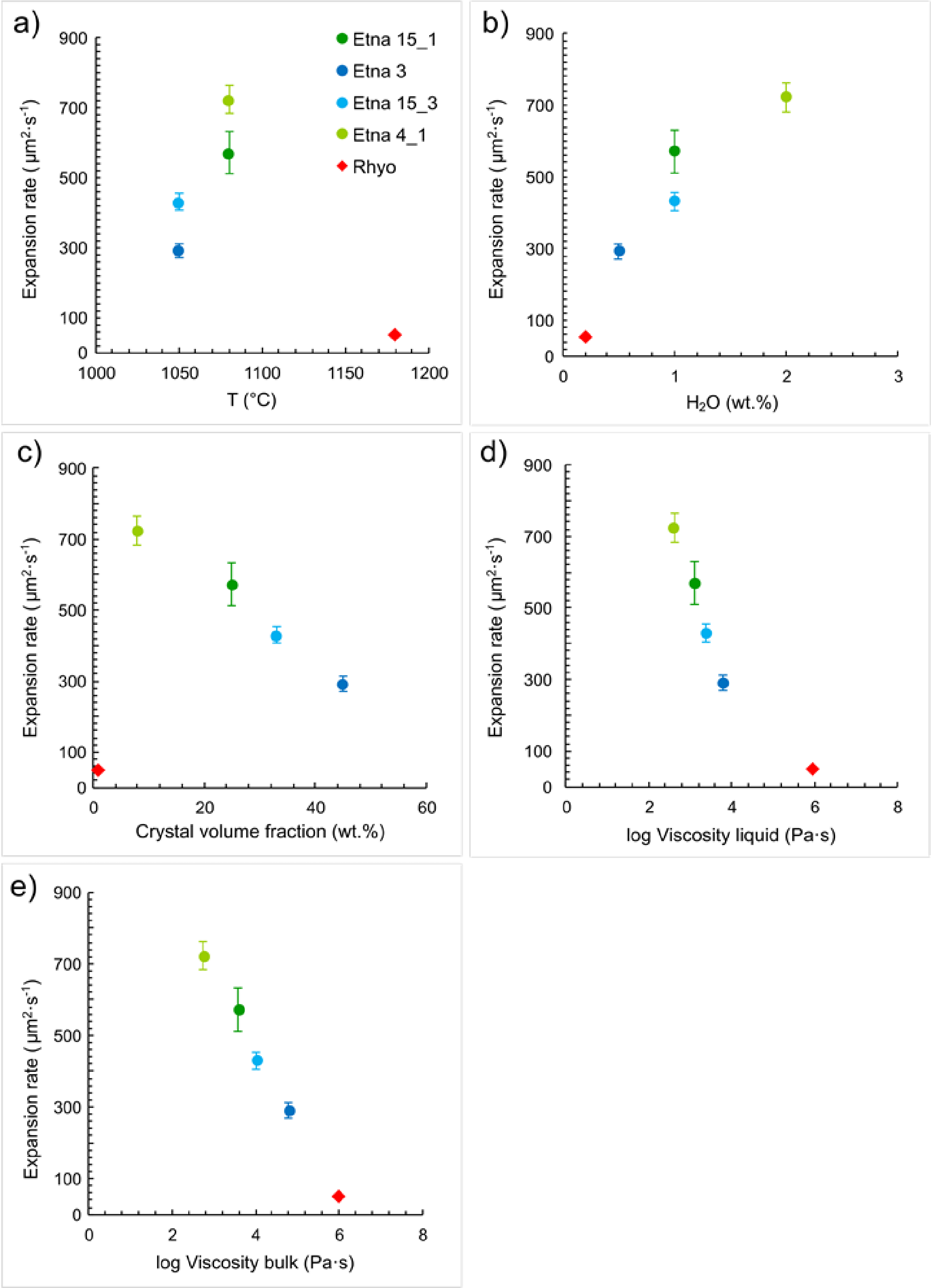

Other Supplementary Materials (not in this PDF)

Table S1. Chemical composition of the starting material

Table S2. Experimental run conditions

Table S3. Average bubble expansion rate parameters

Table S4. Bubble growth rate parameters for Superliq_Dec experiments

Table S5. Numerical results obtained using the bubble growth model of Coumans et al. (2020) assuming different decompression rates, water content and bubble number density

Table S6. Bubble expansion rate parameters

Table S7. Bubble coalescence parameters for Superliq_Dec experiments

Table S8. Ascent velocity and time to surface for basaltic lava fountaining activities on Kilauea

Movie S1. (separate file)

Supplementary movies showing vesiculation during decompression in a-b) Superliq_Dec (Etna12_2, Rhyo) and c) Subliq_Dec (Etna 4_1) runs.

Movie S2. (separate file)

Supplementary movie showing vesiculation during decompression for Etna 12_2 run (Superliq_Dec) together with P and T data.

Movie S3. (separate file)

Supplementary movie showing vesiculation during decompression for Etna 15_3 run (Subliq_Dec) together with P and T data.

REFERENCES AND NOTES

1. R. S. J. Sparks, The dynamics of bubble formation and growth in magmas: A review and analysis. *J. Volcanol. Geotherm. Res.* **3**, 1–37 (1978).

2. Y. Liu, Y. Zhang, H. Behrens, Solubility of H_2O in rhyolitic melts at low pressures and a new empirical model for mixed H_2O–CO_2 solubility in rhyolitic melts. *J. Volcanol. Geotherm. Res.* **143**, 219–235 (2005).

3. A. Toramaru, Measurement of bubble size distributions in vesiculated rocks with implications for quantitative estimation of eruption processes. *J. Volcanol. Geotherm. Res.* **43**, 71–90 (1990).

4. Y. Zhang, Z. Xu, M. Zhu, H. Wang, Silicate melt properties and volcanic eruptions. *Rev. Geophys.* **45**, RG4004 (2007).

5. M. T. Mangan, K. V. Cashman, S. Newman, Vesiculation of basaltic magma during eruption. *Geology* **21**, 157–160 (1993).

6. M. R. Burton, H. M. Mader, M. Polacci, The role of gas percolation in quiescent degassing of persistently active basaltic volcanoes. *Earth Planet. Sci. Lett.* **264**, 46–60 (2007).

7. J. M. Castro, A. Burgisser, C. I. Schipper, S. Mancini, Mechanisms of bubble coalescence in silicic magmas. *Bull. Volcanol.* **74**, 2339–2352 (2012).

8. M. Polacci, R. A. Corsaro, D. Andronico, Coupled textural and compositional characterization of basaltic scoria: Insights into the transition from Strombolian to fire fountain activity at Mount Etna Italy. *Geology* **34**, 201–204 (2006).

9. S. Lovejoy, H. Gaonac'h, D. Schertzer, Bubble distributions and dynamics: The expansion-coalescence equation. *J. Geophys. Res. Solid Earth* **109**, B11203 (2004).

10. H. R. Westrich, J. C. Eichelberger, Gas transport and bubble collapse in rhyolitic magma: An experimental approach. *Bull. Volcanol.* **56**, 447–458 (1994).

11. J. D. Blower, J. P. Keating, H. M. Mader, J. C. Phillips, Inferring volcanic degassing processes from vesicle size distributions. *Geophys. Res. Lett.* **28**, 347–350 (2001).

12. J. C. Eichelberger, C. R. Carrigan, H. R. Westrich, R. H. Price, Non-explosive silicic volcanism. *Nature* **323**, 598–602 (1986).

13. H. M. Gonnermann, M. Manga, The fluid mechanics inside a volcano. *Annu. Rev. Fluid Mech.* **39**, 321–356 (2007).

14. J. E. Gardner, Bubble coalescence in rhyolitic melts during decompression from high pressure. *J. Volcanol. Geotherm. Res.* **166**, 161–176 (2007).

15. T. Giachetti, H. M. Gonnermann, J. E. Gardner, A. Burgisser, S. Hajimirza, T. C. Earley, N. Truong, P. Toledo, Bubble coalescence and percolation threshold in expanding rhyolitic magma. *Geochem. Geophys. Geosyst.* **20**, 1054–1074 (2019).

16. C. Martel, G. Iacono-Marziano, Timescales of bubble coalescence, outgassing, and foam collapse in decompressed rhyolitic melts. *Earth Planet. Sci. Lett.* **412**, 173–185 (2015).

17. D. S. Martula, T. Hasegawa, D. R. Lloyd, R. T. Bonnecaze, Coalescence-induced coalescence of inviscid droplets in a viscous fluid. *J. Colloid Interface Sci.* **232**, 241–253 (2000).

18. C. T. Nguyen, H. M. Gonnermann, Y. Chen, C. Huber, A. A. Maiorano, A. Gouldstone, J. Dufek, Film drainage and the lifetime of bubbles. *Geochem. Geophys. Geosyst.* **14**, 3616–3631 (2013).

19. S. Okumura, M. Nakamura, A. Tsuchiyama, Shear-induced bubble coalescence in rhyolitic melts with low vesicularity. *Geophys. Res. Lett.* **33**, L20316 (2006).

20. A. A. Proussevitch, D. L. Sahagian, Dynamics of coupled diffusive and decompressive bubble growth in magmatic systems. *J. Geophys. Res. Solid Earth* **101**, 17447–17455 (1996).

21. C. Klug, K. V. Cashman, Permeability development in vesiculating magmas: Implications for fragmentation. *Bull. Volcanol.* **58**, 87–100 (1996).

22. O. Navon, V. Lyakhovsky, Vesiculation processes in silicic magmas. *Geol. Soc. Spec. Publ.* **145**, 27–50 (1998).

23. M. Manga, H. A. Stone, Interactions between bubbles in magmas and lavas: Effects of bubble deformation. *J. Volcanol. Geotherm. Res.* **63**, 267–279 (1994).

24. M. Manga, H. A. Stone, Collective hydrodynamics of deformable drops and bubbles in dilute low Reynolds number suspensions. *J. Fluid Mech.* **300**, 231–263 (1995).

25. C. Huber, Y. Su, C. T. Nguyen, A. Parmigiani, H. M. Gonnermann, J. Dufek, A new bubble dynamics model to study bubble growth, deformation, and coalescence. *J. Geophys. Res. Earth* **119**, 216–239 (2014).

26. S. Mancini, L. Forestier-Coste, A. Burgisser, F. James, J. Castro, An expansion–coalescence model to track gas bubble populations in magmas. *J. Volcanol. Geotherm. Res.* **313**, 44–58 (2016).

27. M. Ohashi, T. Maruishi, A. Toramaru, Coalescence of growing bubbles in highly viscous liquids. *Geochem. Geophys. Geosyst.* **23**, e2022GC010618 (2022).

28. M. Ohashi, A. Toramaru, A. Namiki, Coalescence of two growing bubbles in a Hele–Shaw cell. *Sci. Rep.* **12**, 1270 (2022).

29. I. Fortelný, A. Živný, J. JŮza, Coarsening of the phase structure in immiscible polymer blends. Coalescence or Ostwald ripening? *J. Polym. Sci. B Polym. Phys.* **37**, 181–187 (1999).

30. J. F. Larsen, M.-H. Denis, J. E. Gardner, Experimental study of bubble coalescence in rhyolitic and phonolitic melts. *Geochim. Cosmochim. Acta* **68**, 333–344 (2004).

31. A. A. Proussevitch, D. L. Sahagian, V. A. Kutolin, Stability of foams in silicate melts. *J. Volcanol. Geotherm. Res.* **59**, 161–178 (1993).

32. R. A. Herd, H. Pinkerton, Bubble coalescence in basaltic lava: Its impact on the evolution of bubble populations. *J. Volcanol. Geotherm. Res.* **75**, 137–157 (1997).

33. L. Karlstrom, E. M. Dunham, Excitation and resonance of acoustic-gravity waves in a column of stratified, bubbly magma. *J. Fluid Mech.* **797**, 431–470 (2016).

34. J. D. Paulsen, R. Carmigniani, A. Kannan, J. C. Burton, S. R. Nagel, Coalescence of bubbles and drops in an outer fluid. *Nat. Commun.* **5**, 3182 (2014).

35. C. R. Anthony, P. M. Kamat, S. S. Thete, J. P. Munro, J. R. Lister, M. T. Harris, O. A. Basaran, Scaling laws and dynamics of bubble coalescence. *Phys. Rev. Fluids* **2**, 083601 (2017).

36. R. Chen, J. Zeng, H. Yu, Mechanism of damped oscillation in microbubble coalescence. *Comput. Fluids* **183**, 38–42 (2019).

37. R. Chen, H. W. Yu, J. Zeng, L. Zhu, General power-law temporal scaling for unequal-size microbubble coalescence. *Phys. Rev. E* **101**, 023106 (2020).

38. J. E. Gardner, M. Hilton, M. R. Carroll, Experimental constraints on degassing of magma: Isothermal bubble growth during continuous decompression from high pressure. *Earth Planet. Sci. Lett.* **168**, 201–218 (1999).

39. C. Gondé, C. Martel, M. Pichavant, H. Bureau, In situ bubble vesiculation in silicic magmas. *Am. Mineral.* **96**, 111–124 (2011).

40. J. F. Larsen, J. E. Gardner, Experimental constraints on bubble interactions in rhyolite melts: Implications for vesicle size distributions. *Earth Planet. Sci. Lett.* **180**, 201–214 (2000).

41. C. Martel, H. Bureau, In situ high-pressure and high-temperature bubble growth in silicic melts. *Earth Planet. Sci. Lett.* **191**, 115–127 (2001).

42. O. Navon, A. Chekhmir, V. Lyakhovsky, Bubble growth in highly viscous melts: Theory, experiments, and autoexplosivity of dome lavas. *Earth Planet. Sci. Lett.* **160**, 763–776 (1998).

43. J. Barclay, D. S. Riley, R. S. J. Sparks, Analytical models for bubble growth during decompression of high viscosity magmas. *Bull. Volcanol.* **57**, 422–431 (1995).

44. J. P. Coumans, E. W. Llewellin, F. B. Wadsworth, M. C. S. Humphreys, S. A. Mathias, B. M. Yelverton, J. E. Gardner, An experimentally validated numerical model for bubble growth in magma. *J. Volcanol. Geotherm. Res.* **402**, 107002 (2020).

45. A. A. Proussevitch, D. L. Sahagian, Dynamics and energetics of bubble growth in magmas: Analytical formulation and numerical modeling. *J. Geophys. Res. Solid Earth* **103**, 18223–18251 (1998).

46. A. Toramaru, Numerical study of nucleation and growth of bubbles in viscous magmas. *J. Geophys. Res.* **100**, 1913–1931 (1995).

47. A. Toramaru, Vesiculation process and bubble size distributions in ascending magmas with constant velocities. *J. Geophys. Res. Solid Earth* **94**, 17523–17542 (1989).

48. M. Hamada, D. Laporte, N. Cluzel, K. T. Koga, T. Kawamoto, Simulating bubble number density of rhyolitic pumices from Plinian eruptions: Constraints from fast decompression experiments. *Bull. Volcanol.* **72**, 735–746 (2010).

49. N. Le Gall, M. Pichavant, Experimental simulation of bubble nucleation and magma ascent in basaltic systems: Implications for Stromboli volcano. *Am. Mineral.* **101**, 1967–1985 (2016).

50. N. Le Gall, M. Pichavant, Homogeneous bubble nucleation in H_2O- and H_2O-CO_2-bearing basaltic melts: Results of high temperature decompression experiments. *J. Volcanol. Geotherm. Res.* **327**, 604–621 (2016).

51. N. G. Lensky, O. Navon, V. Lyakhovsky, Bubble growth during decompression of magma: Experimental and theoretical investigation. *J. Volcanol. Geotherm. Res.* **129**, 7–22 (2004).

52. M. Mangan, T. Sisson, Delayed, disequilibrium degassing in rhyolite magma: Decompression experiments and implications for explosive volcanism. *Earth Planet. Sci. Lett.* **183**, 441–455 (2000).

53. C. C. Mourtada-Bonnefoi, D. Laporte, Kinetics of bubble nucleation in a rhyolitic melt: An experimental study of the effect of ascent rate. *Earth Planet. Sci. Lett.* **218**, 521–537 (2004).

54. A. Toramaru, BND (bubble number density) decompression rate meter for explosive volcanic eruptions. *J. Volcanol. Geotherm. Res.* **154**, 303–316 (2006).

55. Y. Liu, Y. Zhang, Bubble growth in rhyolitic melt. *Earth Planet. Sci. Lett.* **181**, 251–264 (2000).

56. V. Lyakhovsky, S. Hurwitz, O. Navon, Bubble growth in rhyolitic melts: Experimental and numerical investigation. *Bull. Volcanol.* **58**, 19–32 (1996).

57. N. S. Bagdassarov, D. B. Dingwell, M. C. Wilding, Rhyolite magma degassing: An experimental study of melt vesiculation. *Bull. Volcanol.* **57**, 587–601 (1996).

58. M. Masotta, H. Ni, H. Keppler, In situ observations of bubble growth in basaltic, andesitic and rhyodacitic melts. *Contrib. Mineral. Petrol.* **167**, 1–14 (2014).

59. C. Gondé, D. Massare, H. Bureau, C. Martel, M. Pichavant, R. Clocchiatti, In situ study of magmatic processes: A new experimental approach. *High Press. Res.* **26**, 243–250 (2006).

60. D. R. Baker, F. Brun, L. Mancini, J. L. Fife, A. LaRue, C. O'Shaughnessy, R. J. Hill, M. Polacci, The importance of pore throats in controlling the permeability of magmatic foams. *Bull. Volcanol.* **81**, 54 (2019).

61. D. R. Baker, F. Brun, C. O'Shaughnessy, L. Mancini, J. L. Fife, M. Rivers, A four-dimensional X-ray tomographic microscopy study of bubble growth in basaltic foam. *Nat. Commun.* **3**, 1135 (2012).

62. F. Arzilli, G. La Spina, M. R. Burton, M. Polacci, N. Le Gall, M. E. Hartley, D. Di Genova, B. Cai, N. T. Vo, E. C. Bamber, S. Nonni, R. Atwood, E. W. Llewellin, R. A. Brooker, H. M. Mader, P. D. Lee, Magma fragmentation in highly explosive basaltic eruptions induced by rapid crystallization. *Nat. Geosci.* **12**, 1023–1028 (2019).

63. E. C. Bamber, G. La Spina, F. Arzilli, M. de Michieli Vitturi, M. Polacci, M. E. Hartley, M. Petrelli, J. Fellowes, M. Burton, Basaltic Plinian eruptions at Las Sierras-Masaya volcano driven by cool storage of crystal-rich magmas. *Commun. Earth Environ.* **3**, 253 (2022).

64. E. C. Bamber, F. Arzilli, M. Polacci, M. E. Hartley, J. Fellowes, D. Di Genova, D. Chavarría, J. A. Saballos, M. R. Burton, Pre- and syn-eruptive conditions of a basaltic Plinian eruption at Masaya Volcano, Nicaragua: The Masaya Triple Layer (2.1 ka). *J. Volcanol. Geotherm. Res.* **392**, 106761 (2020).

65. M. Coltelli, P. Del Carlo, L. Vezzoli, Discovery of a Plinian basaltic eruption of Roman age at Etna volcano Italy. *Geology* **26**, 1095 (1998).

66. L. Costantini, B. F. Houghton, C. Bonadonna, Constraints on eruption dynamics of basaltic explosive activity derived from chemical and microtextural study: The example of the Fontana Lapilli Plinian eruption, Nicaragua, *J. Volcanol. Geotherm. Res.* **189**, 207–224 (2010).

67. B. F. Houghton, H. M. Gonnermann, Basaltic explosive volcanism: Constraints from deposits and models. *Geochemistry* **68**, 117–140 (2008).

68. P. Moitra, H. M. Gonnermann, B. F. Houghton, T. Giachetti, Relating vesicle shapes in pyroclasts to eruption styles. *Bull. Volcanol.* **75**, 691 (2013).

69. G. La Spina, F. Arzilli, E. W. Llewellin, M. R. Burton, A. B. Clarke, M. de Michieli Vitturi, M. Polacci, M. E. Hartley, D. Di Genova, H. M. Mader, Explosivity of basaltic lava fountains is controlled by magma rheology, ascent rate and outgassing. *Earth Planet. Sci. Lett.* **553**, 116658 (2021).

70. L. Bai, D. R. Baker, M. Rivers, Experimental study of bubble growth in Stromboli basalt melts at 1 atm. *Earth Planet. Sci. Lett.* **267**, 533–547 (2008).

71. Y. Zhang, H. Ni, Diffusion of H, C, and O components in silicate melts. *Rev. Mineral. Geochem.* **72**, 171–225 (2010).

72. A. Lindoo, An experimental study of permeability development as a function of crystal-free melt viscosity. *Earth Planet. Sci. Lett.* **435**, 45–54 (2016).

73. A. C. Rust, M. Manga, K. V. Cashman, Determining flow type, shear rate and shear stress in magmas from bubble shapes and orientations. *J. Volcanol. Geotherm. Res.* **122**, 111–132 (2003).

74. A. Toramaru, Formation of propagation pattern in two-phase flow systems with application to volcanic eruptions. *Geophys. J. Int.* **95**, 613–623 (1988).

75. D. Giordano, J. K. Russell, D. B. Dingwell, Viscosity of magmatic liquids: A model. *Earth Planet. Sci. Lett.* **271**, 123–134 (2008).

76. M. Polacci, D. R. Baker, L. Mancini, S. Favretto, R. J. Hill, Vesiculation in magmas from Stromboli and implications for normal Strombolian activity and paroxysmal explosions in basaltic systems. *J. Geophys. Res. Solid Earth* **114**, 10.1029/2008JB005672 (2009).

77. L. Bai, D. R. Baker, M. Polacci, R. J. Hill, In-situ degassing study on crystal-bearing Stromboli basaltic magmas: Implications for Stromboli explosions. *Geophys. Res. Lett.* **38**, L17309 (2011).

78. A. Theurel, M. Collombet, A. Burgisser, C. Martel, L. Arbaret, R. Champallier, Experimental evidence of primary permeability at very low gas content in crystal-rich silicic magma. *Geophys. Res. Lett.* **51**, e2024GL108389 (2024).

79. E. A. Parfitt, L. Wilson, Explosive volcanic eruptions-IX. The transition between Hawaiian-style lava fountaining and Strombolian explosive activity. *Geophys. J. Int.* **121**, 226–232 (1995).

80. J. E. Sable, B. F. Houghton, P. Del Carlo, M. Coltelli, Changing conditions of magma ascent and fragmentation during the Etna 122 BC basaltic Plinian eruption: Evidence from clast microtextures. *J. Volcanol. Geotherm. Res.* **158**, 333–354 (2006).

81. S. Vergniolle, C. Jaupart, Separated two-phase flow and basaltic eruptions. *J. Geophys. Res. Solid Earth* **91**, 12842–12860 (1986).

82. P. Papale, A. Neri, G. Macedonio, The role of magma composition and water content in explosive eruptions. *J. Volcanol. Geotherm. Res.* **87**, 75–93 (1998).

83. L. Wilson, R. S. J. Sparks, G. P. L. Walker, Explosive volcanic eruptions—IV. The control of magma properties and conduit geometry on eruption column behaviour. *Geophys. J. Roy. Astron. Soc.* **63**, 117–148 (1980).

84. J. Crozier, S. Tramontano, P. Forte, S. J. C. Oliva, H. M. Gonnermann, E. Lev, M. Manga, M. Myers, E. Rader, P. Ruprecht, H. Tuffen, R. Paisley, B. F. Houghton, T. Shea, C. I. Schipper, J. M. Castro, Outgassing through magmatic fractures enables effusive eruption of silicic magma. *J. Volcanol. Geotherm. Res.* **430**, 107617 (2022).

85. H. M. Gonnermann, M. Manga, Explosive volcanism may not be an inevitable consequence of magma fragmentation. *Nature* **426**, 432–435 (2003).

86. C. Jaupart, Gas loss from magmas through conduit walls during eruption. *Geol. Soc. Lond. Spec. Publ.* **145**, 73–90 (1998).

87. E. A. Parfitt, A discussion of the mechanisms of explosive basaltic eruptions. *J. Volcanol. Geotherm. Res.* **134**, 77–107 (2004).

88. M. Polacci, D. R. Baker, L. Bai, L. Mancini, Large vesicles record pathways of degassing at basaltic volcanoes. *Bull. Volcanol.* **70**, 1023–1029 (2008).

89. E. C. Bamber, G. La Spina, F. Arzilli, M. Polacci, L. Mancini, M. De Michieli Vitturi, D. Andronico, R. A. Corsaro, M. R. Burton, Outgassing behaviour during highly explosive basaltic eruptions. *Commun. Earth Environ.* **5**, 3 (2024).

90. Y. Moussallam, E. F. Rose-Koga, K. T. Koga, E. Médard, P. Bani, J.-L. Devidal, D. Tari, Fast ascent rate during the 2017–2018 Plinian eruption of Ambae (Aoba) volcano: A petrological investigation. *Contrib. Mineral. Petrol.* **174**, 90 (2019).

91. K. Goepfert, J. E. Gardner, Influence of pre-eruptive storage conditions and volatile contents on explosive Plinian style eruptions of basic magma. *Bull. Volcanol.* **72**, 511–521 (2010).

92. P. Moitra, B. F. Houghton, Porosity-permeability relationships in crystal-rich basalts from Plinian eruptions. *Bull. Volcanol.* **83**, 71 (2021).

93. J. Sable, B. Houghton, C. Wilson, R. Carey, "Eruption mechanisms during the climax of the Tarawera 1886 basaltic Plinian eruption inferred from microtextural characteristics of the deposits" in

Studies in Volcanology: The Legacy of George Walker, T. Thordarson, S. Self, G. Larsen, S. K. Rowland, A. Höskuldsson, Eds. (Geological Society, 2009), pp. 129–154.

94. L. A. Szramek, Mafic Plinian eruptions: Is fast ascent required? *J. Geophys. Res. Solid Earth* **121**, 7119–7136 (2016).

95. M. Colombier, J. Vasseur, B. F. Houghton, F. Cáceres, B. Scheu, U. Kueppers, S. Thivet, L. Gurioli, C. Montanaro, A. Soldati, A. Di Muro, D. B. Dingwell, Degassing and gas percolation in basaltic magmas. *Earth Planet. Sci. Lett.* **573**, 117134 (2021).

96. D. J. Ferguson, H. M. Gonnermann, P. Ruprecht, T. Plank, E. H. Hauri, B. F. Houghton, D. A. Swanson, Magma decompression rates during explosive eruptions of Kīlauea volcano, Hawaii, recorded by melt embayments. *Bull. Volcanol.* **78**, 71 (2016).

97. E. Del Bello, E. W. Llewellin, J. Taddeucci, P. Scarlato, S. J. Lane, An analytical model for gas overpressure in slug-driven explosions: Insights into Strombolian volcanic eruptions. *J. Geophys. Res. Solid Earth* **117**, B02206 (2012).

98. E. W. Llewellin, E. Del Bello, J. Taddeucci, P. Scarlato, S. J. Lane, The thickness of the falling film of liquid around a Taylor bubble. *Proc. R. Soc. Math. Phys. Eng. Sci.* **468**, 1041–1064 (2012).

99. D. Carbone, L. Zuccarello, A. Messina, S. Scollo, H. Rymer, Balancing bulk gas accumulation and gas output before and during lava fountaining episodes at Mt. Etna. *Sci. Rep.* **5**, 18049 (2016).

100. M. Cassidy, M. Manga, K. Cashman, O. Bachmann, Controls on explosive-effusive volcanic eruption styles. *Nat. Commun.* **9**, 2839 (2018).

101. H. M. Gonnermann, Magma Fragmentation. *Annu. Rev. Earth Planet. Sci.* **43**, 431–458 (2015).

102. E. W. Llewellin, H. M. Mader, S. D. R. Wilson, The constitutive equation and flow dynamics of bubbly magmas. *Geophys. Res. Lett.* **29**, 2170 (2002).

103. E. W. Llewellin, M. Manga, Bubble suspension rheology and implications for conduit flow. *J. Volcanol. Geotherm. Res.* **143**, 205–217 (2005).

104. C. Oppenheimer, R. Moretti, P. R. Kyle, A. Eschenbacher, J. B. Lowenstern, R. L. Hervig, N. W. Dunbar, Mantle to surface degassing of alkalic magmas at Erebus volcano, Antarctica, *Earth Planet. Sci. Lett.* **306**, 261–271 (2011).

105. P. Valdivia, A. A. Marshall, B. D. Brand, M. Manga, C. Huber, Mafic explosive volcanism at Llaima Volcano: 3D x-ray microtomography reconstruction of pyroclasts to constrain shallow conduit processes. *Bull. Volcanol.* **84**, 2 (2022).

106. A. Burgisser, L. Chevalier, J. E. Gardner, J. M. Castro, The percolation threshold and permeability evolution of ascending magmas. *Earth Planet. Sci. Lett.* **470**, 37–47 (2017).

107. M. Colombier, F. B. Wadsworth, B. Scheu, J. Vasseur, K. J. Dobson, F. Cáceres, A. Allabar, F. Marone, C. M. Schlepütz, D. B. Dingwell, In situ observation of the percolation threshold in multiphase magma analogues. *Bull. Volcanol.* **82**, 32 (2020).

108. F. Arzilli, M. Polacci, G. La Spina, N. Le Gall, E. W. Llewellin, R. A. Brooker, R. Torres-Orozco, D. Di Genova, D. A. Neave, M. E. Hartley, H. M. Mader, D. Giordano, R. Atwood, P. D. Lee, F. Heidelbach, M. R. Burton, Dendritic crystallization in hydrous basaltic magmas controls magma mobility within the Earth's crust. *Nat. Commun.* **13**, 3354 (2022).

109. R. A. Corsaro, L. Miraglia, M. Pompilio, Petrologic evidence of a complex plumbing system feeding the July–August 2001 eruption of Mt. Etna, Sicily, Italy. *Bull. Volcanol.* **69**, 401–421 (2007).

110. N. Le Gall, F. Arzilli, G. La Spina, M. Polacci, B. Cai, M. E. Hartley, N. T. Vo, R. C. Atwood, D. Di Genova, S. Nonni, E. W. Llewellin, M. R. Burton, P. D. Lee, In situ quantification of crystallisation kinetics of plagioclase and clinopyroxene in basaltic magma: Implications for lava flow. *Earth Planet. Sci. Lett.* **568**, 117016 (2021).

111. M. Polacci, F. Arzilli, G. La Spina, N. Le Gall, B. Cai, M. E. Hartley, D. Di Genova, N. T. Vo, S. Nonni, R. C. Atwood, E. W. Llewellin, P. D. Lee, M. R. Burton, Crystallisation in basaltic magmas revealed via in situ 4D synchrotron X-ray microtomography. *Sci. Rep.* **8**, 8377 (2018).

112. F. Menges, Spectragryph - optical spectroscopy software, version 1.2.16 (2022); www.effemm2.de/spectragryph/.

113. D. Testemale, R. Argoud, O. Geaymond, J.-L. Hazemann, High pressure/high temperature cell for x-ray absorption and scattering techniques. *Rev. Sci. Instrum.* **76**, 043905 (2005).

114. R. Bruyère, A. Prat, C. Goujon, J.-L. Hazemann, A new pressure regulation device using high pressure isolation valves. *J. Phys. Conf. Ser.* **121**, 122003 (2008).

115. M. D. Abramoff, P. J. Magalhães, S. J. Ram, Image processing with ImageJ. *Biophotonics Int.* **11**, 36–42 (2004).

116. K. V. Cashman, M. T. Mangan, Physical aspects of magmatic degassing; II, Constraints on vesiculation processes from textural studies of eruptive products. *Rev. Mineral. Geochem.* **30**, 447–478 (1994).

117. O. Sigmarsson, D. Laporte, M. Carpentier, B. Devouard, J.-L. Devidal, J. Marti, Formation of U-depleted rhyolite from a basanite at El Hierro, Canary Islands, *Contrib. Mineral. Petrol.* **165**, 601–622 (2013).

118. M. S. Ghiorso, G. A. R. Gualda, An H_2O–CO_2 mixed fluid saturation model compatible with rhyolite-MELTS. *Contrib. Mineral. Petrol.* **169**, 53 (2015).

119. G. A. R. Gualda, M. S. Ghiorso, R. V. Lemons, T. L. Carley, Rhyolite-MELTS: A modified calibration of MELTS optimized for silica-rich, fluid-bearing magmatic systems. *J. Petrol.* **53**, 875–890 (2012).

120. A. Vona, C. Romano, D. B. Dingwell, D. Giordano, The rheology of crystal-bearing basaltic magmas from Stromboli and Etna. *Geochim. Cosmochim. Acta* **75**, 3214–3236 (2011).

121. A. Burgisser, J. E. Gardner, Experimental constraints on degassing and permeability in volcanic conduit flow. *Bull. Volcanol.* **67**, 42–56 (2004).
